# Control charts for chronic disease surveillance: testing algorithm sensitivity to changes in data coding

**DOI:** 10.1186/s12889-021-12328-w

**Published:** 2022-02-28

**Authors:** Naomi C. Hamm, Depeng Jiang, Ruth Ann Marrie, Pourang Irani, Lisa M. Lix

**Affiliations:** 1 Department of Community Health Sciences, Max Rady College of Medicine, Rady Faculty of Health Sciences, University of Manitoba, S113-750 Bannatyne Avenue, Winnipeg, MB R3E 0W3 Canada; 2 Department of Internal Medicine, Max Rady College of Medicine, Rady Faculty of Health Sciences, University of Manitoba, Winnipeg, MB R3A 1R9 Canada; 3 Department of Computer Science, University of Manitoba, Winnipeg, MB R3T 2N2 Canada

**Keywords:** Control charts, Chronic disease surveillance, International classification of diseases codes, Administrative health data

## Abstract

**Background:**

Algorithms used to identify disease cases in administrative health data may be sensitive to changes in the data over time. Control charts can be used to assess how variations in administrative health data impact the stability of estimated trends in incidence and prevalence for administrative data algorithms. We compared the stability of incidence and prevalence trends for multiple juvenile diabetes algorithms using observed-expected control charts.

**Methods:**

Eighteen validated algorithms for juvenile diabetes were applied to administrative health data from Manitoba, Canada between 1975 and 2018. Trends in disease incidence and prevalence for each algorithm were modelled using negative binomial regression and generalized estimating equations; model-predicted case counts were plotted against observed counts. Control limits were set as predicted case count ±0.8*standard deviation. Differences in the frequency of out-of-control observations for each algorithm were assessed using McNemar’s test with Holm-Bonferroni adjustment.

**Results:**

The proportion of out-of-control observations for incidence and prevalence ranged from 0.57 to 0.76 and 0.45 to 0.83, respectively. McNemar’s test revealed no difference in the frequency of out-of-control observations across algorithms. A sensitivity analysis with relaxed control limits (2*standard deviation) detected fewer out-of-control years (incidence 0.19 to 0.33; prevalence 0.07 to 0.52), but differences in stability across some algorithms for prevalence.

**Conclusions:**

Our study using control charts to compare stability of trends in incidence and prevalence for juvenile diabetes algorithms found no differences for disease incidence. Differences were observed between select algorithms for disease prevalence when using wider control limits.

**Supplementary Information:**

The online version contains supplementary material available at 10.1186/s12889-021-12328-w.

## Background

Administrative health data are widely used for monitoring trends in chronic disease incidence and prevalence for entire populations. Algorithms (i.e., case definitions) to ascertain disease cases may be applied to administrative health data without considering potential changes in the data over time. Specifically, changes in clinical guidelines, diagnosis coding practices, and healthcare processes may impact how administrative health data are coded [1, 2]. Therefore, changes in observed disease trends may reflect changes in data coding rather than true changes in population health status [3–7]. Methods that attempt to disentangle true change from coding-related effects will benefit users of administrative health data for disease surveillance.

Originally developed to monitor industrial processes, control charts are used to graph observed data in sequential order, with a centre line representing the average or expected value [8]. Control limits set around the centre line are used to denote the range where sources of process variation can be attributed to random error. Observations outside the control limits are deemed ‘out-of-control’, suggesting a non-random source of variation influenced the process of interest [8]. Different kinds of control charts can be applied to process data, including Shewhart charts, U′ charts, cumulative sum charts (CUMSU), and observed-expected charts. Chart selection depends on the data characteristics and chart purpose.

There has been a steady uptake of control charts in population health and healthcare research since the 1990s, with a marked increase in recent years [9]. Applications include monitoring: mortality rates using observed-expected [10], CUMSU [11], and p charts [10]; hospital length-of-stay using exponentially weighted moving average [12], CUMSU [12], and Shewhart charts [13]; surgical infection rates using Q [14] and p charts [15]; and delivery outcomes for maternity wards using observed-expected charts [16]. In health surveillance settings, U′ charts have been used to monitor injury rates of military personnel [17] and Shewhart and CUMSU charts have been used to detect changes in child blood lead levels [18]. In addition, open source software has already been developed to apply control charts to infectious disease surveillance using REDCap, R, and the R Shiny package [19].

Risk-adjusted control charts are of particular interest for health surveillance as they can adjust for different risk strata in the population [20, 21]. Risk-adjusted CUMSU and observed-expected control charts, which are closely related and sometimes used interchangeably [11, 21, 22], are risk-adjusted charts commonly used in health research due to their ease of interpretability and versatility with different data types (e.g., binary, count, continuous data) [9, 11, 22]. Risk-adjusted CUMSU charts incorporate observed values from previous time points into control limit calculations [22], whereas observed-expected charts may not.

Control charts could also be used to monitor chronic disease surveillance estimates obtained from administrative health data, similar to their applications to mortality data. Out-of-control diseases estimates may indicate where changes in trends are due to changes in coding practices or other factors affecting the data, rather than true changes in population health. Moreover, comparing control charts across multiple algorithms that use different sources of data (i.e., hospital versus physician records) may help to reveal potential sources of non-random process variation and indicate whether some algorithms are more affected by data variations (i.e., less stable) than other algorithms.

Given this background, the purpose of this study was to apply observed-expected control charts to incidence and prevalence trends in a case study of one disease. The objectives were to a) visualize the stability of disease trends over time; and b) compare the stability of incidence and prevalence trends produced using different algorithms applied to administrative data.

## Methods

### Selection of algorithms

PubMed, Google Scholar, and Embase were searched up to October 2020 for juvenile diabetes algorithms for administrative health data. Juvenile diabetes was selected as the focus of this study because administrative health data have frequently been used for surveillance of this disease and multiple validated algorithms have been developed [23, 24]. Search terms included diabetes, children, juvenile, administrative health data, case definition, claims data, incidence, and prevalence. Only articles published in the English language were reviewed.

**Fig. 1:**
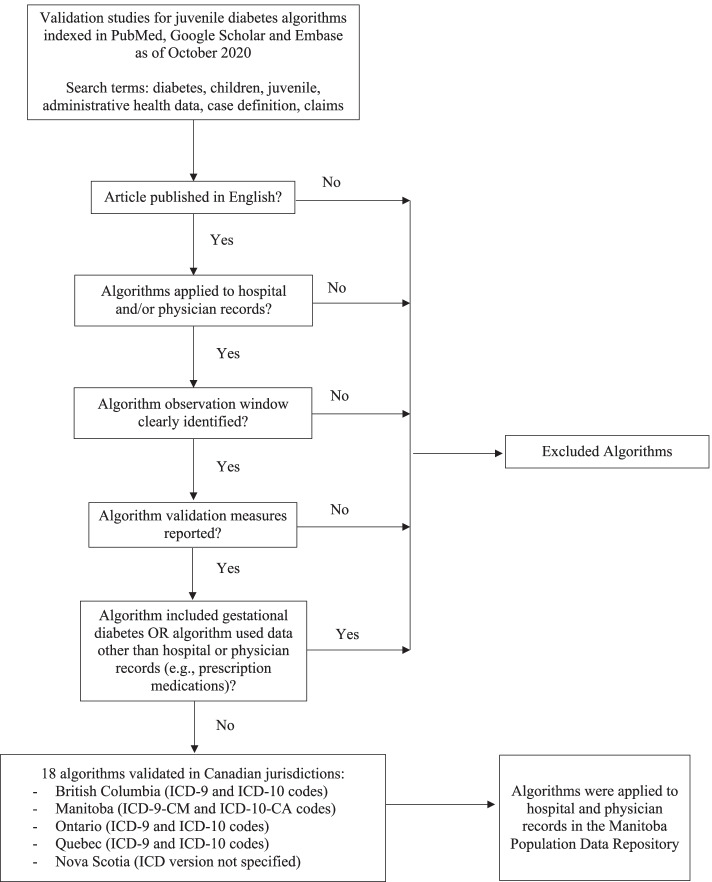
Flowchart of juvenile diabetes algorithm selection from published literature

Algorithms were selected for this study if they used hospital and/or physician records, if the number of records and observation window (i.e., number of years for a diagnosis to occur within the records) for the algorithm was clearly stated, and if validation measures (e.g., sensitivity, specificity) were reported. Algorithms were excluded if they included gestational diabetes or used data other than hospital or physician records, such as prescription medications. We adopted the latter exclusion, because our primary interest was in data coded using International Classification of Disease (ICD) codes. Table 1 summarizes the 18 algorithms we identified from the literature to include in this study [23–29]. Six algorithms were validated in Manitoba, Canada; three were validated in British Columbia, Canada; 13 were validated in Ontario, Canada; 16 were validated in Quebec, Canada; and one was validated in Nova Scotia, Canada. Figure 1 provides a flowchart that describes algorithm selection.

**Table 1:** Validated algorithms used to identify juvenile diabetes cases in administrative health data

Algorithm Name	Algorithm Description	References	ICD Codes
1: 1 + H or 1 + P	1 or more hospital separation or 1 or more physician visit in 1 year	23, 25, 28	ICDA-8: 249, 250 ICD-9-CM: 250.^b^ ICD-CA: E10.^b^ - E14.^b^
1: 1 + H or 2 + P	1 or more hospital separation or 2 or more physician visits in 1 year	23, 25, 28, 29	
1: 1 + H or 3 + P	1 or more hospital separation or 3 or more physician visits in 1 year	23, 28	
1: 1 + H or 4 + P	1 or more hospital separation or 4 or more physician visits in 1 year	28	
2: 1 + H or 1 + P	1 or more hospital separation or 1 or more physician visit in 2 years	23, 25	
2: 1 + H or 2 + P	1 or more hospital separation or 2 or more physician visits in 2 years	23, 24, 25, 26, 27, 28	
3: 1 + H or 1 + P	1 or more hospital separation or 1 or more physician visit in 3 years	25	
3: 1 + H or 2 + P	1 or more hospital separation or 2 or more physician visits in 3 years	25, 28	
1: 1 + P	1 or more physician visits in 1 year	23, 28	
1: 2 + P	2 or more physician visits in 1 year^a^	23, 28	
1: 3 + P	3 or more physician visits in 1 year^a^	23, 28	
1: 4 + P	4 or more physician visits in 1 year^a^	23, 28	
1: 5 + P	5 or more physician visits in 1 year^a^	23, 28	
2: 1 + P	1 or more physician visits in 2 years	23, 28	
2: 2 + P	2 or more physician visits in 2 years^a^	23, 28	
2: 3 + P	3 or more physician visits in 2 years^a^	23, 28	
2: 4 + P	4 or more physician visits in 2 years^a^	23, 24, 28	
2: 5 + P	5 or more physician visits in 2 years^a^	23, 28	

^a^Visits must be at least 30 days apart, ^b^ includes all codes beginning with specified digits

### Data source

Algorithms were applied to data from the Manitoba Population Data Repository housed at the Manitoba Centre for Health Policy (MCHP). The study period was Jan 1, 1972 to Dec 31, 2018. Manitoba has a universal healthcare system and a population of 1.3 million residents. The Manitoba Health Insurance Registry, Hospital Discharge Abstracts, and Medical Claims/Medical Services databases were used. The Manitoba Health Insurance Registry contains health insurance coverage dates, birth date, and sex. The Hospital Discharge Abstracts and Medical Claims/Medical Services databases contain ICD codes and dates for hospital and physician visits, respectively. Three ICD versions are used to code diagnoses within these two databases: ICD Adapted (A)-8, ICD-9-Clinical Modifications (CM), and ICD-10-Canadian version (CA). For hospital visits captured in Hospital Discharge Abstracts, records between January 1, 1972 and March 31, 1979 are coded using 4-digit ICDA-8 codes; records between April 1, 1979 and March 31, 2004 are coded using 5-digit ICD-9-CM codes; and records from April 1, 2004 onwards are coded using 5-digit ICD-10-CA codes. For physician visits captured in Medical Claims/Medical Services, records between January 1, 1972 and March 31, 1979 are coded using ICDA-8 and records from April 1, 1979 onwards are coded using ICD-9-CM. Diagnosis codes for physician visits are recorded at the 3-digit level until March 31, 2015 (both ICDA-8 and ICD-9-CM); 5-digit codes are used from April 1, 2015 onward (ICD-9-CM). For both databases, data from 1972 to 1974 were originally collected using using the 7th revision of ICD codes and later converted to ICDA-8 by the data provider. These years were not included in the study analysis because we had no information about the conversion method used by the data provider. However, data from these years were used to establish the lookback period for defining incident cases (see Study cohort and study periods).

Incident and prevalent disease counts per year were aggregated by sex and age group (0-9 years; 10-17 years). Cell sizes less than six were suppressed, as per provincial health privacy regulations.

### Study cohort and study periods

Separate cohorts were created for each algorithm. To be included in a study cohort, individuals required continuous health insurance coverage during the observation window (1 to 3 years, depending on the algorithm). Individuals in each cohort were classified as cases if they met the criteria of the respective algorithm. A 3 year look back period was used for incidence [23], meaning only individuals with no diabetes claims in the prior 3 years were identified as incident cases.

Study ICD periods were defined based on the ICD version that was used at the beginning of each year. There were three ICD periods: ICDA-8 (1975 to 1979), ICD-9 (1980 to 2004), and ICD-9/10 (2005 to 2018). ICD implementation periods were defined as the 2 years before, after, and including the year a new ICD version was implemented. There were two ICD implementation periods: ICDA-8 to -9 (1977 to 1981) and ICD-9 to -9/10 (2002 to 2006).

### Statistical analysis

The estimated annual crude rate per 100,000 population was calculated; this was the number of cases per year divided by the number of individuals with continuous healthcare coverage per year, multiplied by 100,000. An average rate was calculated for each ICD period; this was the average value of the annual crude rates in that time period. The average annual rate of change in each ICD period was calculated as the total change in crude rate (annual crude rate in the last year of the ICD period minus the annual crude rate in the first year of the ICD period) divided by the number of years in the ICD period.

For each algorithm, incident case counts, where observations for successive years are independent (i.e., not correlated), were modelled using negative binomial regression models. Prevalent case counts, where observations for successive years are correlated, were modelled using generalized estimating equation (GEE) models that assume a Poisson distribution; this GEE produces correct estimates of the population average model parameters (i.e., prevalence) and their standard errors in the presence of dependence between repeated observations. The GEE model adopted a first order autoregressive correlation structure because the data modelled were time series data. For all models, age group, sex, and year were included as covariates. The natural logarithm of the cohort size was defined as the model offset. To account for potential non-linear effects of year, the shape of the year effect was tested using a restricted cubic spline [30]. Four models were applied to the data: one with year as a linear effect, and three with year as a restricted cubic spline with three, four, and five knots, respectively. Knots were placed at quintiles as recommended by Harrell [30]. The model with year as a restricted cubic spline with the lowest Akaike Information Criterion (AIC) [31] or Quasi Information Criterion (QIC) value [32] was selected as the best fitting model and compared to the model with year as a linear term using a likelihood ratio test (incidence) or Wald test (prevalence). If the test indicated the model with the restricted cubic spline did not fit the data significantly better than the model with year as a linear term (i.e., *p* < .05), the linear model was adopted.

Model fit for the best fitting model was assessed by calculating the residual deviance to degrees of freedom ratio (negative binomial models) or the marginal *R*^2^ values based on Zheng [33] (GEE models). If the number of suppressed cells for an algorithm was greater than 10%, the data were not modelled. If no more than 10% of the cells were suppressed, suppressed cells were randomly imputed to have a value between one and five. Three algorithms had more than 10% of cells suppressed for incidence and one algorithm had more than 10% of cells suppressed for prevalence. Therefore, incidence counts were modelled for 15 of the identified algorithms and prevalence counts were modelled for 17 of the identified algorithms.

Observed-expected control charts were applied by graphing model-predicted counts from the best fitting model against the observed case counts [21, 34]. Predicted values for each year, age group, and sex combination were calculated, along with their respective standard deviations. To obtain a single estimate and standard deviation (SD) for each year during the study period, predicted values were summed across groups and SDs were pooled. Control limits were calculated based on Cohen’s effect size [35] as the model-predicted value ±0.8*pooled SD. This cut-off was chosen as it provided a meaningful understanding of results (i.e., detect large differences between model predicted and observed counts) and did not incorporate a grand mean into the calculation. More information on the calculation of control limits, expected values, and SDs can be found in Additional file 1.

To compare trend stability across algorithms, annual case counts were classified as ‘in-control’ or ‘out-of-control’ for the years 1975 to 2016 based on the calculated control limits. Data after 2016 were truncated, because algorithms with three-year observation windows did not have case counts beyond 2016. Data before 1975 were used to establish the lookback period for defining incident cases. The proportion of out-of-control years was calculated as the total number of out-of-control years for an algorithm divided by the number of study years (i.e., 1975-2016; 42 years).

McNemar’s test [36] was used to test for differences in the frequency of out-of-control observations between algorithms. McNemar’s test was chosen because all algorithms were applied to the same population (i.e., repeated measurements). The algorithm of one or more hospital or physician visits in a two-year period (2: 1 + H or 1 + P) was selected as the reference algorithm, as the literature review identified it as having the highest validation measures (validated using chart abstraction) and was the most common algorithm identified through the literature search. Out-of-control observations for the remaining algorithms were then compared to the reference algorithm to determine differences in trend stability. Trend stability was compared across the entire study period, the three ICD periods, and the two ICD implementation periods. To control the overall probability of a Type I error for each family of tests (i.e., entire study period, each ICD period, and each ICD implementation period), a Holm-Bonferroni adjustment [37] was used. This adjustment controls the Type I error rate, but is more powerful than the traditional Bonferroni adjustment to detect a difference [37, 38].

To identify years that were frequently flagged as out-of-control, an agreement-by-year measure was calculated for each year of the study observation period. This was the total number of algorithms that classified a particular year as out-of-control, divided by the total number of algorithms modelled (i.e., 15 for incidence; 17 for prevalence).

In a sensitivity analysis the control limits were set as the model-predicted value ±2*pooled SD. All data analyses were performed using R version 4.1.0. The MASS package [39] was used to fit the negative binomial models and the geepack package [40] was used to fit the GEE models. All research was performed in accordance with the relevant guidelines and regulations.

## Results

Average crude incidence and prevalence rates and average annual rates of change for each ICD period are reported in Table 2. For both incidence and prevalence, the average crude rate increased from the ICDA-8 period to the ICD-9/10 period, with the exception of the 1: 4 + P algorithm, where prevalence decreased from 39.86 cases per 100,000 population in the ICDA-8 period to 38.30 per 100,000 population in the ICD-9 period. As expected, the average crude rate was lower for algorithms that required more diagnosis codes to identify cases and was higher for algorithms with longer observation windows. All algorithms had a positive average annual crude rate of change for both incidence and prevalence during the ICD-9 and ICD-9/10 periods, except for the algorithm 3: 1 + H or 1 + P, which had a negative average annual crude rate of change for incidence during the ICD-9/10 period. The direction of the average annual crude rate of change was variable across algorithms for the ICDA-8 period.

**Table 2:** Average crude incidence and prevalence rates and average annual rate of change per 100,000 population across ICD periods

Algorithm	ICDA-8 Period (1975-1979)		ICD-9 Period (1980-2004)		ICD-9/10 Period (2005 onwards)^**a**^	
	Average Rate	Average Annual Rate Change	Average Rate	Average Annual Rate Change	Average Rate	Average Annual Rate Change
Incidence						
1: 1 + H or 1 + P	87.38	−0.32	98.31	2.36	134.80	0.67
1: 1 + H or 2 + P	26.76	−0.79	29.22	0.99	51.69	1.93
1: 1 + H or 3 + P	18.22	0.32	23.01	0.67	40.47	1.57
1: 1 + H or 4 + P	16.70	0.23	20.07	0.75	35.74	1.39
2: 1 + H or 1 + P	175.38	−9.46	200.75	4.63	276.18	1.30
2: 1 + H or 2 + P	54.72	−3.87	62.85	1.72	114.45	3.85
3: 1 + H or 1 + P	261.95	−12.38	309.36	6.74	424.79	−0.58
3: 1 + H or 2 + P	82.45	−5.05	100.45	2.68	182.26	5.03
1: 1 + P	86.50	−0.01	97.59	2.32	133.88	0.61
1: 2 + P	12.80	0.67	16.69	0.82	35.40	1.56
2: 1 + P	173.77	−8.84	199.43	4.57	274.02	1.21
2: 2 + P	31.97	−1.29	43.25	1.40	89.44	3.52
2: 3 + P	22.44	0.20	31.46	1.12	64.31	2.40
2: 4 + P	16.69	0.05	24.11	0.92	47.83	2.10
2: 5 + P	12.77	0.39	17.07	0.70	34.10	1.88
Prevalence						
1: 1 + H or 1 + P	180.09	0.47	213.67	5.49	349.73	5.58
1: 1 + H or 2 + P	104.60	−0.55	127.97	3.77	244.72	6.51
1: 1 + H or 3 + P	85.42	−0.63	99.48	2.40	202.88	6.56
1: 1 + H or 4 + P	73.62	0.09	74.61	1.38	142.33	5.81
2: 1 + H or 1 + P	276.62	−9.59	323.62	7.92	499.01	5.63
2: 1 + H or 2 + P	146.55	−4.22	177.70	4.82	322.96	7.96
3: 1 + H or 1 + P	366.82	−13.33	435.26	10.00	651.70	4.65
3: 1 + H or 2 + P	178.73	−5.12	219.52	5.85	394.79	9.48
1: 1 + P	178.64	1.09	212.60	5.43	348.53	5.47
1: 2 + P	84.18	1.37	111.15	3.46	223.79	6.12
1: 3 + P	61.25	1.53	74.05	2.27	168.32	5.44
1: 4 + P	39.86	1.05	38.30	0.56	79.70	3.61
2: 1 + P	274.52	−8.66	322.03	7.86	496.59	5.54
2: 2 + P	120.45	−1.41	155.94	4.47	295.29	7.61
2: 3 + P	101.23	0.27	134.93	4.10	260.28	6.53
2: 4 + P	85.69	2.64	114.84	3.62	231.71	6.32
2: 5 + P	73.78	2.56	90.84	3.02	198.22	6.56

^a^Until 2016 for algorithms with a three-year observation window; 2017 for algorithms with a two-year observation window; 2018 for algorithms with a one-year observation window

### Models

Goodness-of-fit measures for the best fitting negative binomial and GEE models for each algorithm are reported in Table 3. Residual deviance to residual degrees of freedom ratio ranged from 1.04 to 1.23 for the negative binomial regression models; marginal *R*^2^ ranged from 0.83 to 0.98 for the GEE models. All algorithms for juvenile diabetes indicated a non-linear effect of year for incidence and prevalence (i.e., the model with year as a restricted cubic spline was selected as the best fitting model).

**Table 3:** Goodness of fit statistics for negative binomial regression and generalized estimating equation models applied to cases ascertained by juvenile diabetes algorithms

Algorithm	Model Fit Measures			Number of RCS knots
Incidence				
	Residual Deviance	Residual DOF	Residual Deviance/ Residual DOF	
1: 1 + H or 1 + P	175	169	1.04	5
1: 1 + H or 2 + P	178	170	1.05	4
1: 1 + H or 3 + P	182	171	1.06	3
1: 1 + H or 4 + P	192	170	1.13	4
2: 1 + H or 1 + P	172	165	1.04	5
2: 1 + H or 2 + P	173	166	1.04	4
3: 1 + H or 1 + P	167	161	1.04	5
3: 1 + H or 2 + P	168	162	1.04	4
1: 1 + P	175	169	1.04	5
1: 2 + P	192	170	1.13	4
2: 1 + P	171	165	1.04	5
2: 2 + P	182	166	1.10	4
2: 3 + P	186	167	1.11	3
2: 4 + P	192	167	1.15	3
2: 5 + P	205	167	1.23	3
Prevalence				
	Marginal *R*^2^			
1: 1 + H or 1 + P	0.94			5
1: 1 + H or 2 + P	0.97			5
1: 1 + H or 3 + P	0.96			5
1: 1 + H or 4 + P	0.90			5
2: 1 + H or 1 + P	0.92			5
2: 1 + H or 2 + P	0.97			5
3: 1 + H or 1 + P	0.91			5
3: 1 + H or 2 + P	0.97			5
1: 1 + P	0.94			5
1: 2 + P	0.97			5
1: 3 + P	0.96			5
1: 4 + P	0.83			5
2: 1 + P	0.92			5
2: 2 + P	0.98			5
2: 3 + P	0.97			5
2: 4 + P	0.97			5
2: 5 + P	0.96			5

*DOF* Degrees of freedom

### Control charts

**Fig. 2:**
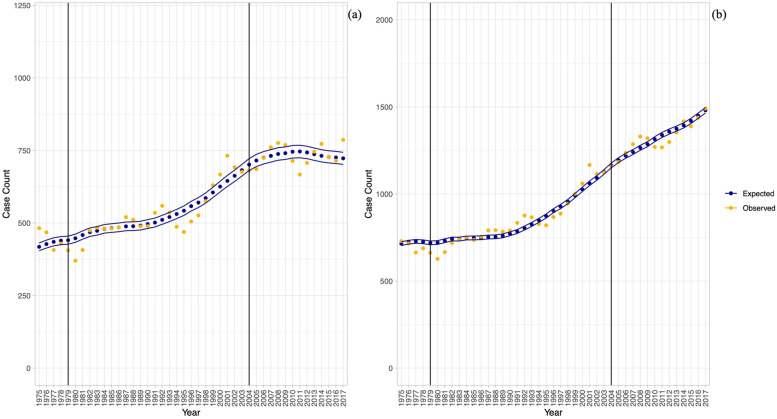
Observed-expected control charts for juvenile diabetes algorithm ‘one or more hospital or physician visits in two years’. Panel **a** shows results for incidence; panel **b** shows results for prevalence. Vertical lines indicate years where a change in ICD version was implemented

Figure 2 shows the observed-expected control charts for incidence and prevalence trends obtained for the reference algorithm (2: 1 + H or 1 + P). Both incidence and prevalence increased over time; the rate of increase was variable over time. The variance (i.e., range) of observed values around expected values was greater for incidence than for prevalence; the control limits for incidence were wider than the control limits for prevalence. Control charts for all algorithms are found in Additional file 2: Figs. S1 and S2.

Table 4 contains information about the proportion of out-of-control years for each algorithm, for both incidence and prevalence. The proportion of out-of-control years ranged from 0.57 to 0.76 for incidence and 0.45 to 0.83 for prevalence. For incidence, the algorithm 2: 5 + P had the greatest proportion of out-of-control years; 2: 3 + P had the lowest proportion of out-of-control years. For prevalence, the algorithm 1: 3 + P had the greatest proportion of out-of-control years and 2: 3 + P had the lowest proportion of out-of-control years.

**Table 4:** Comparisons of incidence and prevalence trend stability across juvenile diabetes algorithms^a,b^

Algorithm	All years (1975-2016)				ICDA-8 Period (1975-1979)		ICD-9 Period (1980-2004)		ICD-9/10 Period (2005-2016)		ICD-8 to − 9 Implementation Period (1977-1981)		ICD-9 to − 9/10 Implementation Period (2002-2006)	
	OOC Count	OOC Prop^c^	***McNemar’s Test***		***McNemar’s Test***		***McNemar’s Test***		***McNemar’s Test***		***McNemar’s Test***		***McNemar’s Test***	
			*p*-value	adj. *p*-value	*p*-value	adj. *p*-value	*p*-value	adj. *p*-value	*p*-value	adj. *p*-value	*p*-value	adj. *p*-value	*p*-value	adj. *p*-value
Incidence														
1: 1 + H or 1 + P	30	0.71	0.48	1.00	1.00	1.00	0.34	1.00	1.00	1.00	1.00	1.00	1.00	1.00
1: 1 + H or 2 + P	29	0.69	0.63	1.00	0.50	1.00	0.15	1.00	1.00	1.00	1.00	1.00	0.50	1.00
1: 1 + H or 3 + P	28	0.67	0.83	1.00	0.25	1.00	0.10	1.00	0.68	1.00	1.00	1.00	1.00	1.00
1: 1 + H or 4 + P	26	0.62	1.00	1.00	0.63	1.00	0.42	1.00	0.68	1.00	1.00	1.00	0.50	1.00
2: 1 + H or 1 + P	26	0.62	REF	REF	REF	REF	REF	REF	REF	REF	REF	REF	REF	REF
2: 1 + H or 2 + P	29	0.69	0.61	1.00	0.50	1.00	**0.02**	0.33	0.68	1.00	1.00	1.00	0.50	1.00
3: 1 + H or 1 + P	29	0.69	0.58	1.00	1.00	1.00	0.68	1.00	1.00	1.00	1.00	1.00	1.00	1.00
3: 1 + H or 2 + P	27	0.64	1.00	1.00	1.00	1.00	0.29	1.00	0.68	1.00	1.00	1.00	1.00	1.00
1: 1 + P	29	0.69	0.65	1.00	1.00	1.00	0.34	1.00	1.00	1.00	1.00	1.00	1.00	1.00
1: 2 + P	29	0.69	0.63	1.00	1.00	1.00	0.50	1.00	1.00	1.00	0.13	1.00	0.50	1.00
2: 1 + P	27	0.64	1.00	1.00	1.00	1.00	1.00	1.00	NA	NA	1.00	1.00	1.00	1.00
2: 2 + P	28	0.67	0.80	1.00	1.00	1.00	0.34	1.00	1.00	1.00	1.00	1.00	0.50	1.00
2: 3 + P	24	0.57	0.81	1.00	0.13	1.00	1.00	1.00	0.68	1.00	0.13	1.00	0.50	1.00
2: 4 + P	27	0.64	1.00	1.00	1.00	1.00	0.55	1.00	0.68	1.00	1.00	1.00	0.50	1.00
2: 5 + P	32	0.76	0.21	1.00	1.00	1.00	0.11	1.00	1.00	1.00	1.00	1.00	0.50	1.00
Prevalence														
1: 1 + H or 1 + P	30	0.71	0.80	1.00	1.00	1.00	1.00	1.00	0.62	1.00	0.13	1.00	1.00	1.00
1: 1 + H or 2 + P	30	0.71	0.80	1.00	0.63	1.00	0.29	1.00	0.13	1.00	0.25	1.00	0.50	1.00
1: 1 + H or 3 + P	34	0.81	0.79	1.00	0.13	1.00	1.00	1.00	1.00	1.00	1.00	1.00	0.63	1.00
1: 1 + H or 4 + P	29	0.69	0.63	1.00	1.00	1.00	0.58	1.00	1.00	1.00	0.13	1.00	1.00	1.00
2: 1 + H or 1 + P	32	0.76	REF	REF	REF	REF	REF	REF	REF	REF	REF	REF	REF	REF
2: 1 + H or 2 + P	25	0.60	0.17	1.00	1.00	1.00	0.72	1.00	0.18	1.00	1.00	1.00	1.00	1.00
3: 1 + H or 1 + P	33	0.79	1.00	1.00	1.00	1.00	0.07	1.00	0.13	1.00	1.00	1.00	0.50	1.00
3: 1 + H or 2 + P	22	0.52	0.06	0.77	1.00	1.00	0.39	1.00	0.08	1.00	0.13	1.00	1.00	1.00
1: 1 + P	30	0.71	0.81	1.00	1.00	1.00	1.00	1.00	0.45	1.00	1.00	1.00	0.63	1.00
1: 2 + P	29	0.69	0.65	1.00	1.00	1.00	0.77	1.00	1.00	1.00	0.13	1.00	1.00	1.00
1: 3 + P	35	0.83	0.61	1.00	1.00	1.00	0.75	1.00	1.00	1.00	0.25	1.00	0.63	1.00
1: 4 + P	31	0.74	1.00	1.00	0.13	1.00	0.58	1.00	1.00	1.00	1.00	1.00	0.50	1.00
2: 1 + P	31	0.74	1.00	1.00	1.00	1.00	NA	NA	1.00	1.00	1.00	1.00	1.00	1.00
2: 2 + P	24	0.57	0.14	1.00	0.25	1.00	0.77	1.00	0.45	1.00	0.25	1.00	1.00	1.00
2: 3 + P	19	0.45	**0.02**	0.26	0.13	1.00	0.39	1.00	0.18	1.00	0.50	1.00	1.00	1.00
2: 4 + P	21	0.50	**0.03**	0.44	0.50	1.00	0.27	1.00	0.22	1.00	0.50	1.00	1.00	1.00
2: 5 + P	26	0.62	0.24	1.00	0.50	1.00	0.58	1.00	1.00	1.00	0.25	1.00	0.50	1.00

*p*-values <.05 are bolded

*OOC* Out-of-control, *Prop* Proportion, *Adj. p-value* Adjusted *p*-value. Adjusted using the Holm-Bonferroni Adjustment Methods

^a^If number of observations < 10, McNemar’s exact test was used. Otherwise McNemar’s approximate test was used

^b^Value of NA indicates results were the same as the comparison group

^c^OOC count over forty-two

McNemar’s test with the Holm-Bonferroni correction found no significant differences in the stability of trends for the reference algorithm compared to other algorithms. The same finding was observed for analyses stratified by ICD period and ICD implementation period.

**Fig. 3:**
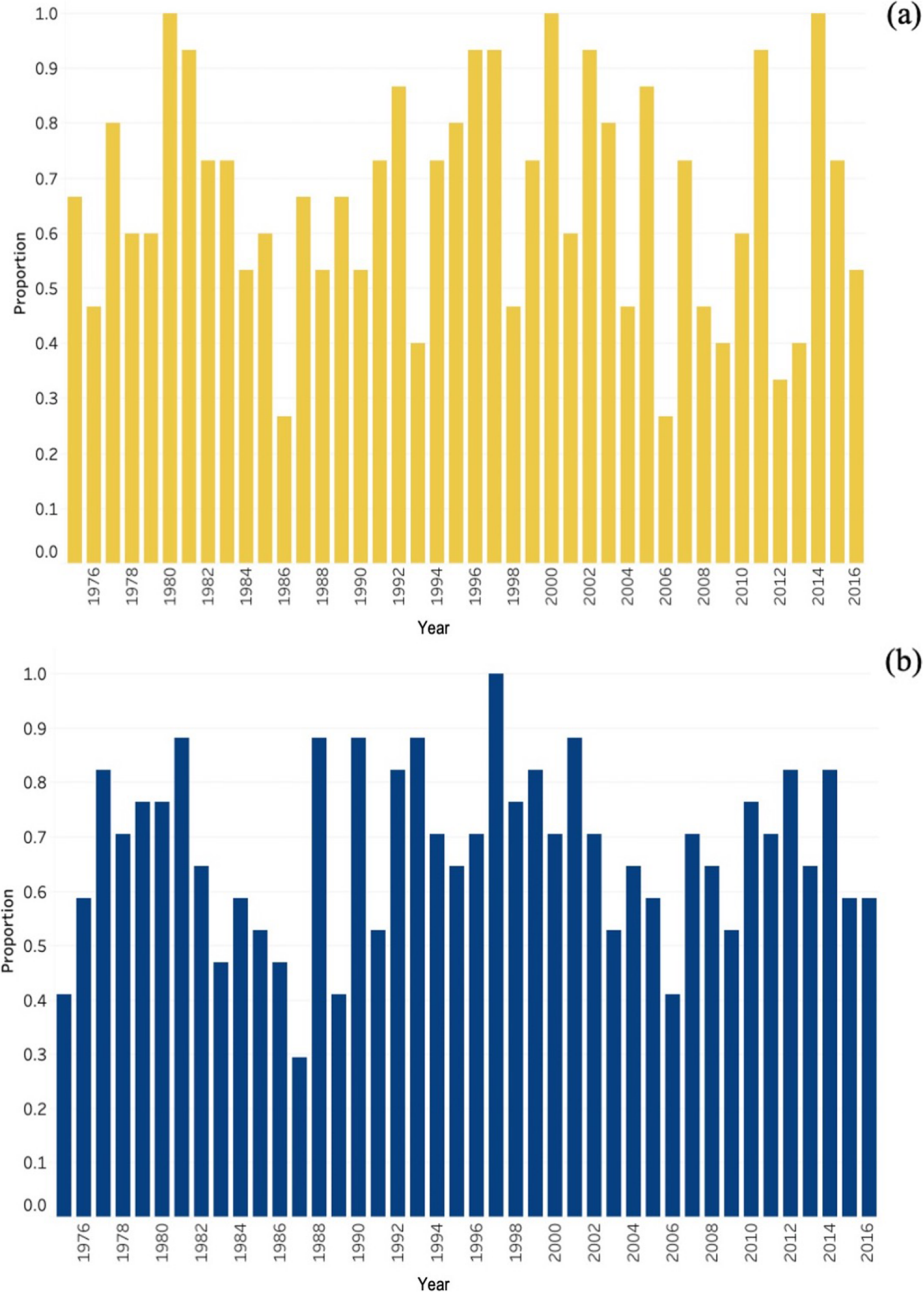
Algorithm agreement-by-year for out-of-control juvenile diabetes estimates. Panel **a** shows results for incidence; panel **b** shows results for prevalence

Figure 3 reports agreement-by-year. For incidence, the years 1980, 2000, and 2004 were flagged as out-of-control for all algorithms. In contrast, 1986 and 2006 were flagged as out-of-control for only four of 15 algorithms. For prevalence, the year 1997 was flagged as out-of-control for all algorithms. The years 1981, 1988, 1990, 1993, and 2001 were flagged as out-of-control for 15 of 17 algorithms. In contrast, 1987 was flagged as out-of-control for only five algorithms.

### Sensitivity analysis

Sensitivity analysis with control limits set at model-predicted value ±2*pooled SD flagged fewer years as out-of-control (Table 5). Control charts for all algorithms can be found in Additional file 2: Figs. S3 and S4. The proportion of out-of-control years ranged from 0.19 to 0.33 for incidence and 0.07 to 0.52 for prevalence. For incidence, the algorithm 2: 3 + P had the lowest proportion of out-of-control years. Two algorithms had the highest proportion of out-of-control years for incidence: 2: 2 + P and 1: 1 + H or 3 + P. For prevalence, the algorithm 2: 2 + P had the lowest proportion and the algorithm 1: 1 + H or 4 + P had the highest proportion of out-of-control years.

**Table 5:** Sensitivity analysis: comparisons of incidence and prevalence trend stability across juvenile diabetes algorithms^a,b^

Algorithm	All years (1975-2016)				ICDA-8 Period (1975-1979)		ICD-9 Period (1980-2004)		ICD-9/10 Period (2005-2016)		ICD-8 to − 9 Implementation Period (1977-1981)		ICD-9 to − 9/10 Implementation Period (2002-2006)	
	OOC Count	OOC Prop^c^	***McNemar’s Test***		***McNemar’s Test***		***McNemar’s Test***		***McNemar’s Test***		***McNemar’s Test***		***McNemar’s Test***	
			*p*-value	adj. *p*-value	*p*-value	adj. *p*-value	*p*-value	adj. *p*-value	*p*-value	adj. *p*-value	*p*-value	adj. *p*-value	*p*-value	adj. *p*-value
Incidence														
1: 1 + H or 1 + P	11	0.26	1.00	1.00	0.50	1.00	1.00	1.00	1.00	1.00	1.00	1.00	1.00	1.00
1: 1 + H or 2 + P	12	0.29	1.00	1.00	0.25	1.00	1.00	1.00	0.48	1.00	1.00	1.00	0.50	1.00
1: 1 + H or 3 + P	14	0.33	0.81	1.00	0.25	1.00	0.77	1.00	0.25	1.00	0.50	1.00	0.25	1.00
1: 1 + H or 4 + P	12	0.29	1.00	1.00	0.25	1.00	0.77	1.00	1.00	1.00	1.00	1.00	0.25	1.00
2: 1 + H or 1 + P	12	0.29	REF	REF	REF	REF	REF	REF	REF	REF	REF	REF	REF	REF
2: 1 + H or 2 + P	12	0.29	1.00	1.00	1.00	1.00	1.00	1.00	1.00	1.00	1.00	1.00	0.25	1.00
3: 1 + H or 1 + P	11	0.26	1.00	1.00	1.00	1.00	1.00	1.00	NA	NA	1.00	1.00	1.00	1.00
3: 1 + H or 2 + P	13	0.31	1.00	1.00	1.00	1.00	1.00	1.00	1.00	1.00	1.00	1.00	1.00	1.00
1: 1 + P	11	0.26	1.00	1.00	0.50	1.00	1.00	1.00	1.00	1.00	1.00	1.00	1.00	1.00
1: 2 + P	12	0.29	1.00	1.00	0.63	1.00	1.00	1.00	0.25	1.00	1.00	1.00	1.00	1.00
2: 1 + P	10	0.24	0.48	1.00	1.00	1.00	1.00	1.00	NA	NA	1.00	1.00	1.00	1.00
2: 2 + P	14	0.33	0.81	1.00	1.00	1.00	1.00	1.00	0.62	1.00	1.00	1.00	0.25	1.00
2: 3 + P	8	0.19	0.45	1.00	1.00	1.00	0.34	1.00	1.00	1.00	1.00	1.00	0.50	1.00
2: 4 + P	11	0.26	1.00	1.00	0.25	1.00	1.00	1.00	0.62	1.00	0.50	1.00	0.50	1.00
2: 5 + P	12	0.29	1.00	1.00	0.25	1.00	1.00	1.00	0.62	1.00	1.00	1.00	0.25	1.00
Prevalence														
1: 1 + H or 1 + P	13	0.31	0.12	1.00	0.63	1.00	0.29	1.00	1.00	1.00	0.50	1.00	1.00	1.00
1: 1 + H or 2 + P	7	0.17	**0.01**	0.10	0.63	1.00	0.10	0.96	0.13	1.00	1.00	1.00	1.00	1.00
1: 1 + H or 3 + P	21	0.50	1.00	1.00	1.00	1.00	1.00	1.00	0.62	1.00	1.00	1.00	0.50	1.00
1: 1 + H or 4 + P	22	0.52	0.82	1.00	0.50	1.00	1.00	1.00	0.13	1.00	0.50	1.00	0.25	1.00
2: 1 + H or 1 + P	20	0.48	REF	REF	REF	REF	REF	REF	REF	REF	REF	REF	REF	REF
2: 1 + H or 2 + P	5	0.12	**< 0.00**	**0.01**	0.50	1.00	**0.01**	0.11	0.22	1.00	0.50	1.00	1.00	1.00
3: 1 + H or 1 + P	22	0.52	0.68	1.00	1.00	1.00	0.68	1.00	NA	NA	1.00	1.00	1.00	1.00
3: 1 + H or 2 + P	9	0.21	**0.01**	0.10	1.00	1.00	**0.02**	0.33	0.45	1.00	0.25	1.00	1.00	1.00
1: 1 + P	13	0.31	0.12	1.00	0.63	1.00	0.29	1.00	1.00	1.00	0.50	1.00	1.00	1.00
1: 2 + P	5	0.12	**< 0.00**	**0.05**	0.63	1.00	0.06	0.80	0.07	1.00	1.00	1.00	1.00	1.00
1: 3 + P	20	0.48	1.00	1.00	1.00	1.00	1.00	1.00	0.62	1.00	1.00	1.00	0.13	1.00
1: 4 + P	20	0.48	1.00	1.00	0.25	1.00	1.00	1.00	0.13	1.00	1.00	1.00	0.25	1.00
2: 1 + P	21	0.50	1.00	1.00	1.00	1.00	NA	NA	1.00	1.00	1.00	1.00	1.00	1.00
2: 2 + P	3	0.07	**< 0.00**	**0.01**	0.25	1.00	**< 0.00**	**0.04**	0.45	1.00	1.00	1.00	1.00	1.00
2: 3 + P	5	0.12	**< 0.00**	**0.05**	0.25	1.00	0.06	0.80	0.22	1.00	1.00	1.00	1.00	1.00
2: 4 + P	5	0.12	**< 0.00**	**0.05**	0.25	1.00	0.06	0.80	0.22	1.00	1.00	1.00	1.00	1.00
2: 5 + P	17	0.40	0.65	1.00	0.50	1.00	0.79	1.00	1.00	1.00	0.50	1.00	0.13	1.00

*p*-values <.05 are in boldface font

Control limits set at 2*SD

*OOC* Out-of-control, *Prop* Proportion, *Adj. p-value* Adjusted *p*-value using the Holm-Bonferroni method

^a^If number of observations < 10, McNemar’s exact test was used. Otherwise McNemar’s approximate test was used

^b^Value of NA indicates results were the same as the comparison group

^c^OOC count over forty-two

For incidence, McNemar’s test revealed no significant differences in trend stability across algorithms (Table 5). For prevalence, differences in trend stability were revealed between the reference algorithm and 2: 1 + H or 2 + P (*p* = 0.010), 1: 2 + P (*p* = 0.049), 2: 2 + P (*p* = 0.008), 2: 3 + P (*p* = 0.049), and 2: 4 + P (*p* = 0.049), where these algorithms had a lower frequency of out-of-control observations. When stratified by ICD period, there was a difference between the reference algorithm and 2: 2 + P (*p* = 0.041) for the ICD-9 period, with 2: 2 + P having a lower frequency of out-of-control observations.

Algorithm agreement-by-year for the sensitivity analysis are reported in Additional file 2: Fig. S5. Incidence counts for the year 1980 was flagged as out-of-control for 14 out of 15 algorithms; prevalence counts for the year 2001 were flagged as out-of-control for 13 out of 17 algorithms.

## Discussion

Observed-expected control charts applied to juvenile diabetes algorithms for administrative health data were used to investigate the stability of trends in incidence and prevalence over a 42-year period in which three ICD versions were used for diagnosis codes. The proportion of out-of-control years detected using control limits of 0.8*SD ranged from 0.57 to 0.76 for incidence and 0.45 to 0.83 for prevalence. As expected, these proportions were reduced to 0.19 to 0.33 for incidence and 0.07 to 0.52 for prevalence when control limits of 2*SD were used in a sensitivity analysis. No differences in trend stability across algorithms were observed in the main analysis. Sensitivity analyses identified five algorithms that produced a more stable prevalence trend compared to the reference algorithm.

Control limits in this analysis were set to have practical meaning and detect a large difference between the observed and expected values, relative to the distribution of the observed data. Applying control limits based on meaningful cut offs has been done before [11, 34]. Wider control limits used in the sensitivity analysis found few changes to the overall study outcome. Previous research that used a similar observed-expected control chart on hospital mortality data indicated poor specificity for control limits larger than 2*SD [34]. Our sensitivity analysis allows users to compare study control limits, while maintaining reasonable specificity for defining an out-of-control observations. Control limits of 2*SD have been used previously when applying control charts to health data [10, 34, 41]. Other potential approaches to set control limits include using a clinical database as the in-control reference or applying a validated algorithm and correcting for potential misclassification rates [42]. The former method requires a population-based clinical database to use as the reference, which may not always be available or accessible.

Our tests of statistical significance did not detect any differences between algorithm agreement of out-of-control years for the main analysis, indicating there was no difference in stability of trends ascertained by different algorithms when compared to a reference algorithm. In contrast, the sensitivity analysis indicated there were five algorithms with a more stable prevalence trend, compared to the reference algorithm. Observation window and data source (i.e., hospital versus physician visits) did not appear to influence differences in observed trend stability. Results from the sensitivity analysis suggest some algorithms are more stable to changes in the coding process when estimating prevalence trends, but only when the specificity for detecting out-of-control years is lower.

Agreement-by-year indicated several years (e.g., 1980, 1981, and 2001) where all, or the majority of algorithms produced an out-of-control estimate in both the main and sensitivity analysis. Previous research examining incidence disease trends over time has called for more studies to examine factors that influence disease trends [43]. The years identified here could provide a starting point to identify those factors. For example, 1980 and 1981 being out-of-control for the majority of algorithms is likely indicative of changes in coding patterns due to the switch from ICDA-8 to ICD-9-CM in 1979, rather than true changes in population health.

This analysis applied control charts to assess the stability of trends over time. While data quality was not directly assessed, trend stability has been used to assess data quality [44]. This is of particular interest, as administrative health data were not originally collected for research and surveillance, potentially impacting the data’s ‘fitness-for-use’. Previous research has used administrative health data in control charts; however, the primary interest was the quality of the healthcare process, not the data itself. Control charts have been used to monitor the quality of cancer registry data [45]; thus, there is a precedent for using control charts as a first step to investigating potential sources of systematic error in administrative data.

### Strengths and limitations

Strengths of this study include the use of observed-expected control charts to assess trend stability. With this method, underlying risk strata were accounted for and calculation of control limits were appropriate to surveillance data (i.e., no grand mean incorporated and therefore appropriate for data trending over time; does not rely on previous case counts). In addition, using restricted cubic splines to model change over time relaxed the assumption of a linear effect without overfitting and reducing the control chart’s ability to detect out-of-control observations. Good model fit was confirmed by the goodness-of-fit measures for the best fitting models.

There are some limitations to this study. While control limits were set to have practical meaning, the accuracy for detecting true out-of-control estimates based on these limits was not tested. To account for this, multiple control limits were used, with the limits for the sensitivity analysis being based on previous literature that used simulations to maximized out-of-control detection accuracy [10, 34].

Clinical data were not used to produce a known ‘in-control’ (i.e., not influenced by error in the data coding process) trend. Rather, a reference algorithm validated using chart abstraction was the comparator for the remaining algorithms. This provided an indication of how trend stability for remaining algorithms compared to a proxy in-control process; however, results may differ when using a clinical database as the standard as defining an in-control reference process.

## Conclusions and future research

Control charts can be used to visualize the stability of chronic disease surveillance trends captured using administrative health data and indicate where potential systematic sources of error may affect surveillance estimates. Differences in trend stability across algorithms were observed for prevalence, but only at wider control limits. Potential areas of future research include identifying optimal control limits for trends ascertained with administrative health data. Future research should also apply control charts to other chronic disease surveillance estimates. Adaptation of control charts as a visual tool to inform policy and decision makers is also a potential area for future research.

## Supplementary Information

**Additional file 1.** Creating Control Limits for Negative Binomial Models Using Cohen’s d. Document describing how control limits were calculated using Cohen’s d.

**Additional file 2.** Control Charts for Main Analysis and Sensitivity Analysis Figures. Control charts for all algorithms from main and sensitivity analysis, as well as algorithm agreement-by-year for the sensitivity analysis.

## Data Availability

The data used in this article was derived from administrative health data as secondary use and provided to the investigators under specific data sharing agreements only for approved use at the Manitoba Centre for Health Policy (MCHP). The original source data is not owned by the researchers or MCHP and as such cannot be provided to a public repository. Where necessary, source data specific to this article or project may be reviewed at MCHP with the consent of the original data providers, along with the required privacy and ethical review bodies. The original data source and approval for use has been noted in the acknowledgments of the article.
