# Supplementary material for: Control charts for chronic disease surveillance: testing algorithm sensitivity to changes in data coding
Source: BMC Public Health. 2022 Feb 28;22:406. doi: 10.1186/s12889-021-12328-w (PMC8883735; doi:10.1186/s12889-021-12328-w)
Supplement: Supplementary file 1 — Additional file 1. Creating Control Limits for Negative Binomial Models Using Cohen’s d. Document describing how control limits were calculated using Cohen’s d. [file 12889_2021_12328_MOESM1_ESM.docx]

**Creating Control Limits for Negative Binomial Models Using Cohen’s d**

Let

Y_ij_ = observed number of cases in year *i* for group *j*, where group represents an age/sex combination

E(Y_ij_) = expected number of counts in year *i* for group *j*, based on the negative binomial model

Y_i_ = total observed cases for year *i*, calculated as $\sum Y_{ij}$

E(Y_i_) = Total expected number of cases for year *i*, calculated as $\sum E{(Y}_{ij})$

- Here the summing of values is appropriate as it takes into account the mixture of risk characteristics (i.e., age and sex groups) in the population

Aim is to see where there are large differences between Y_i_ and E(Y_i_) – large difference determined using Cohen’s d = 0.8

$$d=\frac{(Mean of experimental group)-(Mean of control group)}{Standard Deviation}$$

Where *d=* effect size.

Therefore, let *d*=0.8

Mean of experimental group = Y_i_

Mean of control group = E(Y_i_)

Standard Deviation (SD) = pooled standard deviation of E(Y_i_)

Therefore,

$$0.8=\frac{Y_{i}-{E(Y}_{i})}{Pooled SD {E(Y}_{i})}$$

Which can be re-written as,

$$Y_{i}={E(Y}_{i})+0.8*Pooled SD {E(Y}_{i})$$

If Y_i_ is outside the range of ${E(Y}_{i})\pm0.8*Pooled SD {E(Y}_{i})$, then there is a large difference between the predicted trend/process and the observed trend/process

**Calculating pooled Standard Deviation of E(Y_i_)**

For every combination of input values, possible output values have a mean and variance.

Predicted E(Y_i_) = mean E(Y_i_). SD is the square root of variance

*For incidence*, SD can be calculated using a negative binomial model distribution, where:

Mean = $\mu$, and

variance = $\mu+ \mu^{2}/\theta$, where $\theta$= dispersion parameter

Therefore, SD for each E(Y_ij_) can be determined as √E(Y_ij_)+ E(Y_ij_)^2^/θ

- θ is a constant estimated based on the negative binomial model

*For prevalence*, SD can be calculated using a Poisson distribution, where:

Mean = $\mu$, and

variance = $\mu$

Therefore, SD for each E(Y_ij_) can be determined as √E(Y_ij_)

Once SD for each E(Y_ij_) is calculated, pooled SD for E(Y_i_) can be calculated
