# Supplementary material for: Control charts for chronic disease surveillance: testing algorithm sensitivity to changes in data coding
Source: BMC Public Health. 2022 Feb 28;22:406. doi: 10.1186/s12889-021-12328-w (PMC8883735; doi:10.1186/s12889-021-12328-w)
Supplement: Supplementary file 2 — Additional file 2. Control Charts for Main Analysis and Sensitivity Analysis Figures. Control charts for all algorithms from main and sensitivity analysis, as well as algorithm agreement-by-year for the sensitivity analysis. [file 12889_2021_12328_MOESM2_ESM.docx]

**Control Charts for Main Analysis and Sensitivity Analysis Figures**

**Figure S1. Observed-expected control charts for incidence trends of identified juvenile diabetes algorithms.** (a) algorithm 1: 1+H or 1+P; (b) algorithm 1: 1+H or 2+P; (c) algorithm 1: 1+H or 3+P; (d) algorithm 1: 1+H or 4+P; (e) algorithm 2: 1+H or 1+P; (f) algorithm 2: 1+H or 2+P; (g) algorithm 3: 1+H or 1+P; (h) algorithm 3: 1+H or 2+P; (i) algorithm 1: 1+P; (j) algorithm 1: 2+P; (k) algorithm 2: 1+P; (l) algorithm 2: 2+P; (m) algorithm 2: 3+P; (n) algorithm 2: 4+P; (o) algorithm 2: 5+P

**
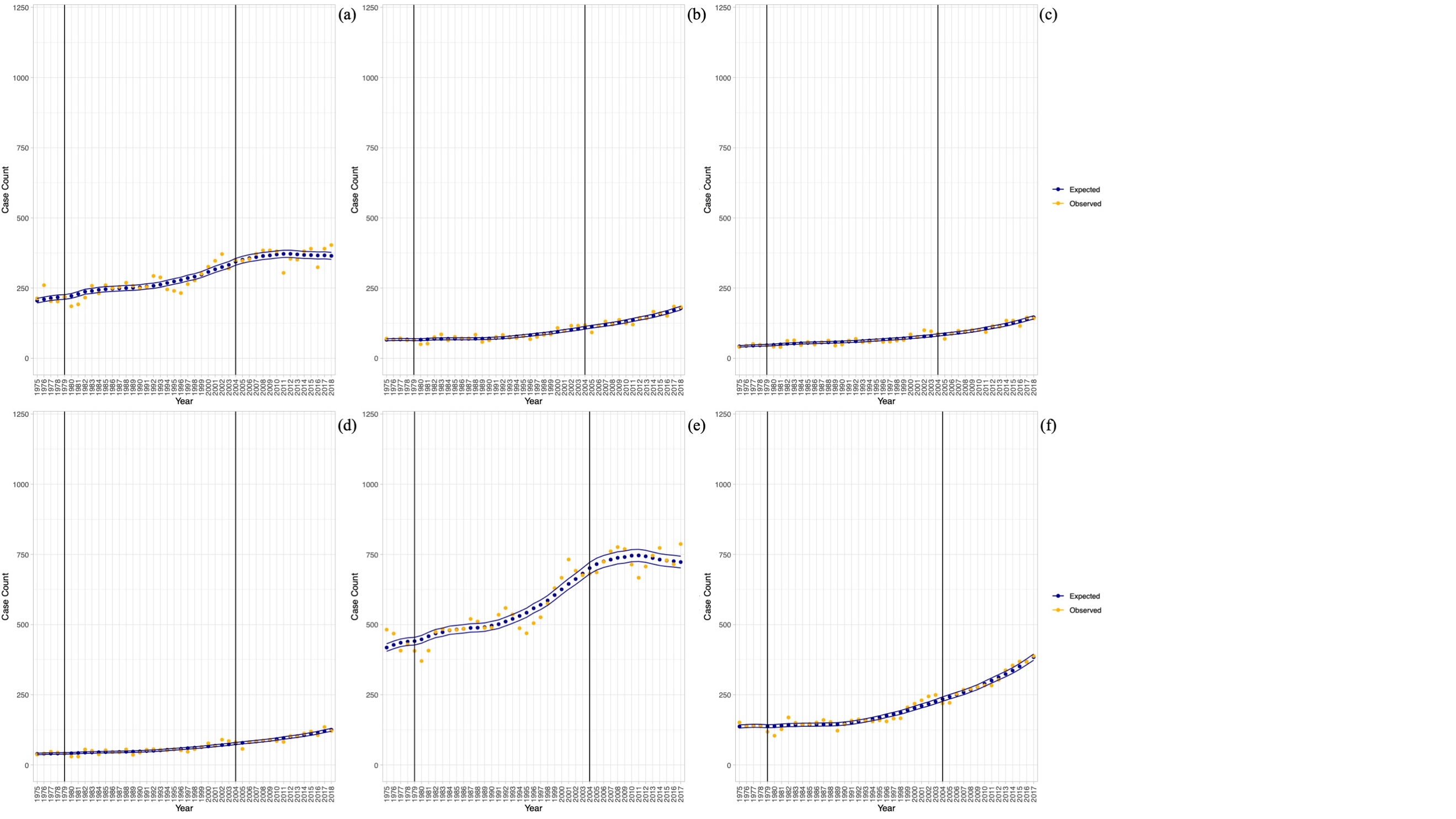
**

**
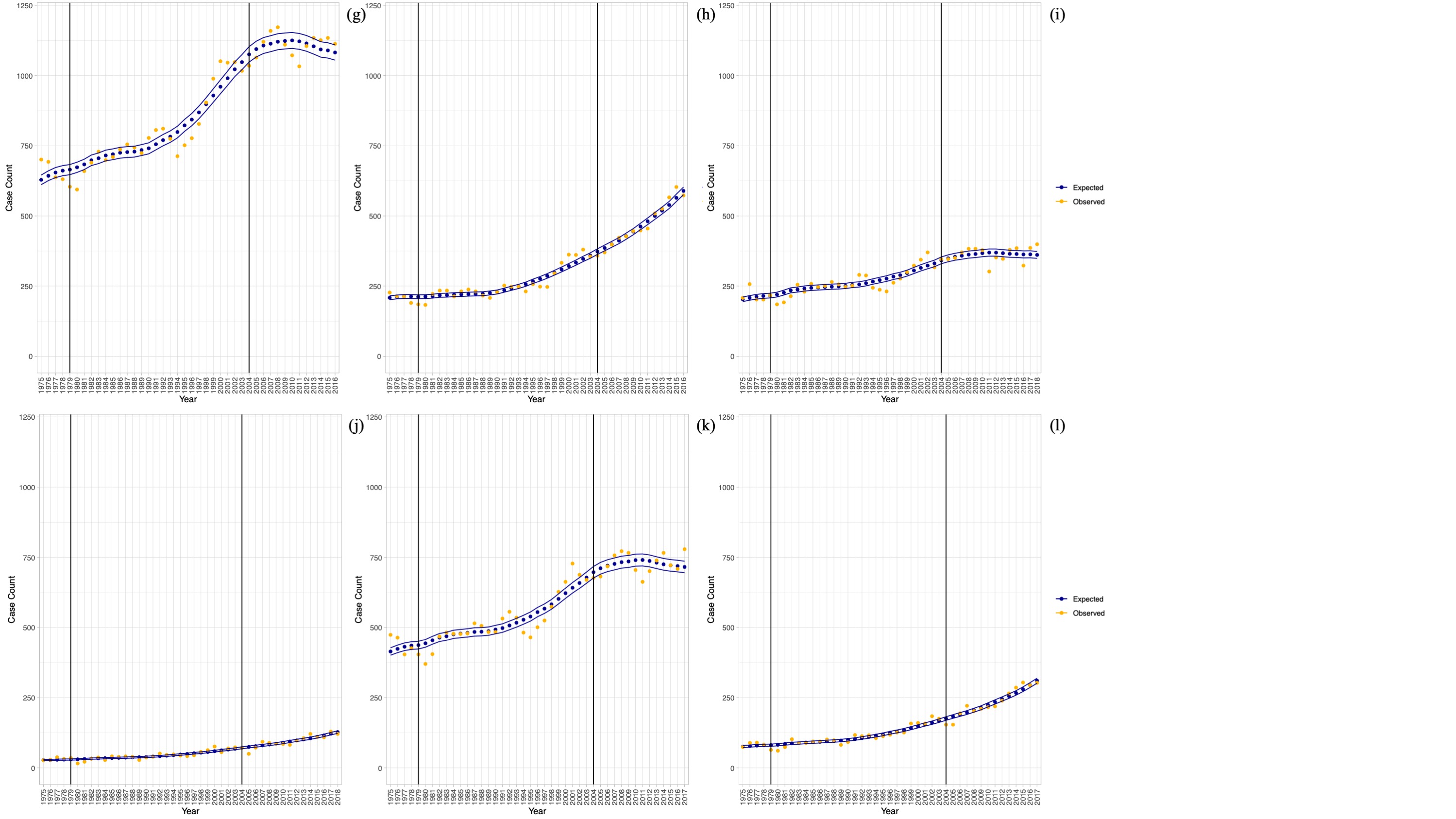
**

**
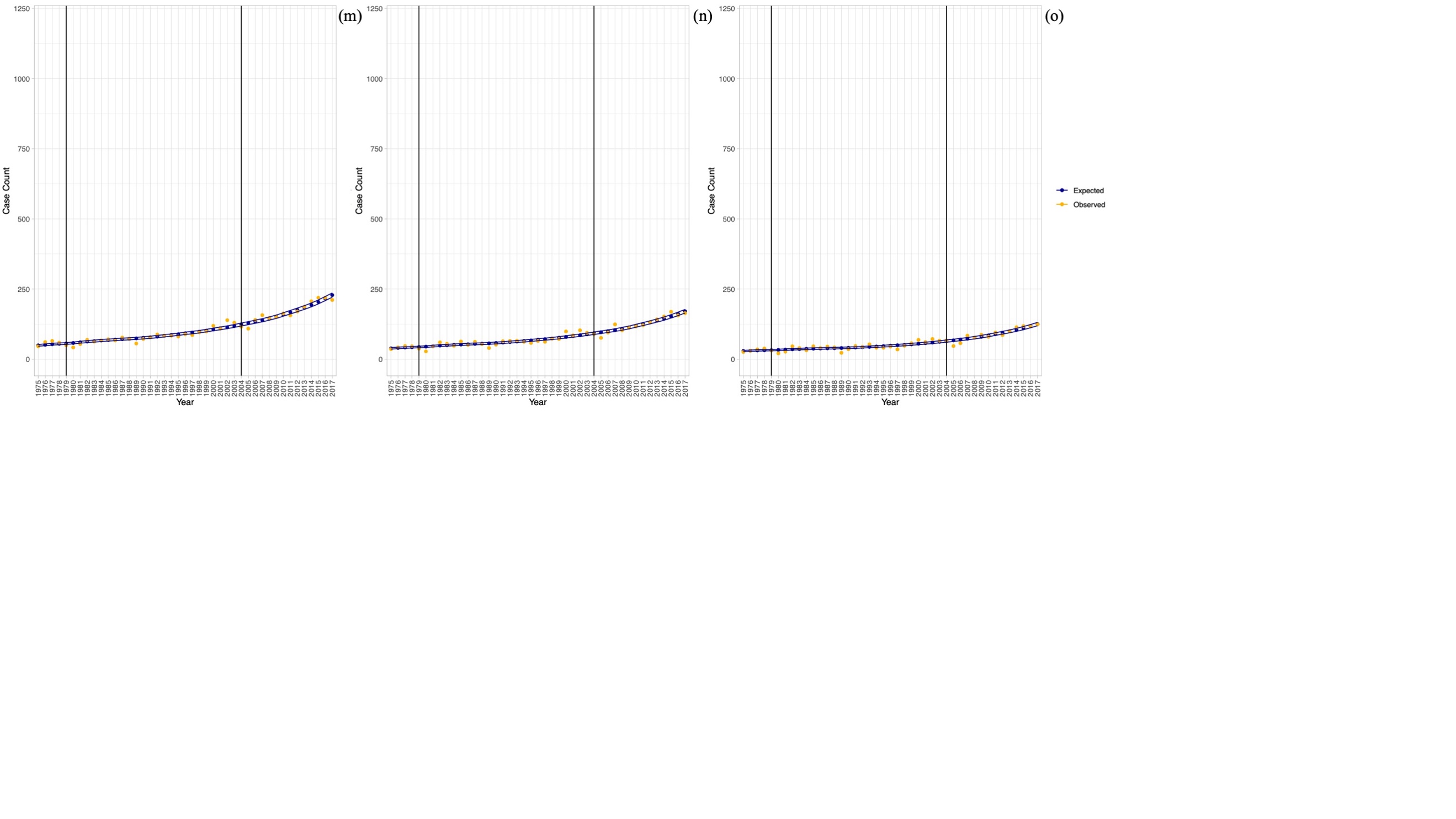
**

**Figure S2. Observed-expected control charts for prevalence trends of identified juvenile diabetes algorithms.** (a) algorithm 1: 1+H or 1+P; (b) algorithm 1: 1+H or 2+P; (c) algorithm 1: 1+H or 3+P; (d) algorithm 1: 1+H or 4+P; (e) algorithm 2: 1+H or 1+P; (f) algorithm 2: 1+H or 2+P; (g) algorithm 3: 1+H or 1+P; (h) algorithm 3: 1+H or 2+P; (i) algorithm 1: 1+P; (j) algorithm 1: 2+P; (k) algorithm 1: 3+P; (l) algorithm 1: 4+P; (m) algorithm 2: 1+P; (n) algorithm 2: 2+P; (o) algorithm 2: 3+P; (p) algorithm 2: 4+P; (q) algorithm 2: 5+P

**
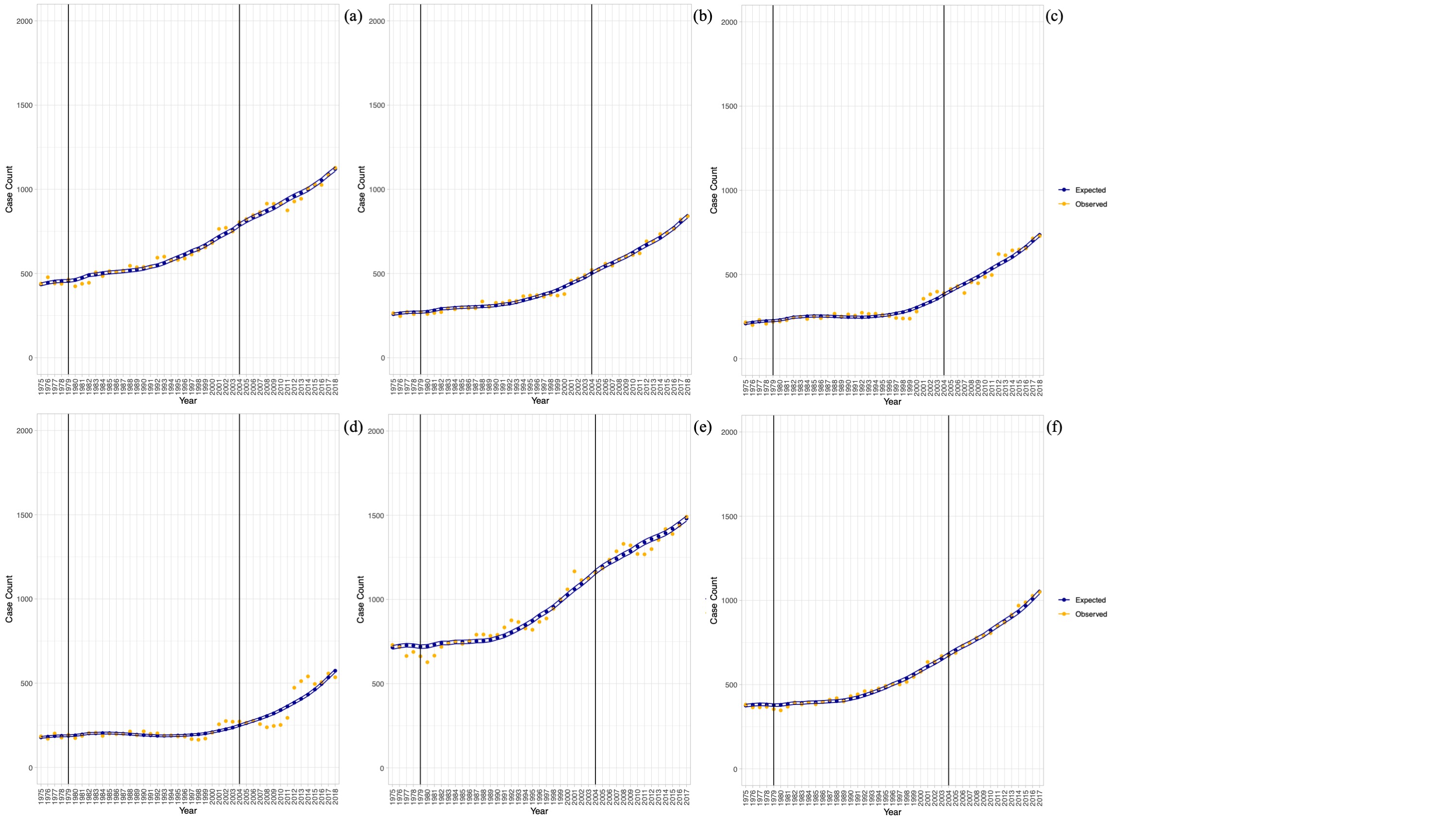
**

**
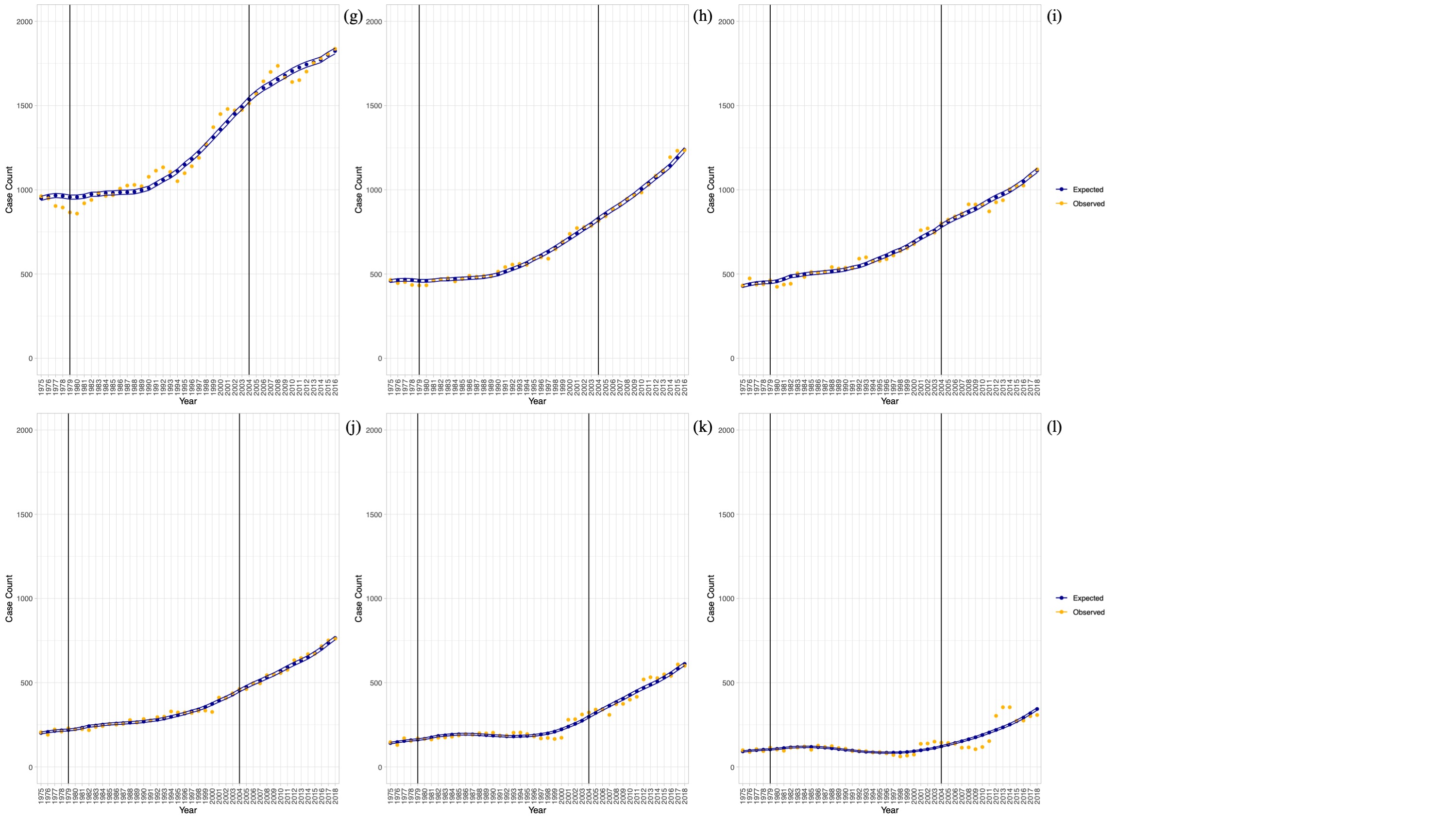

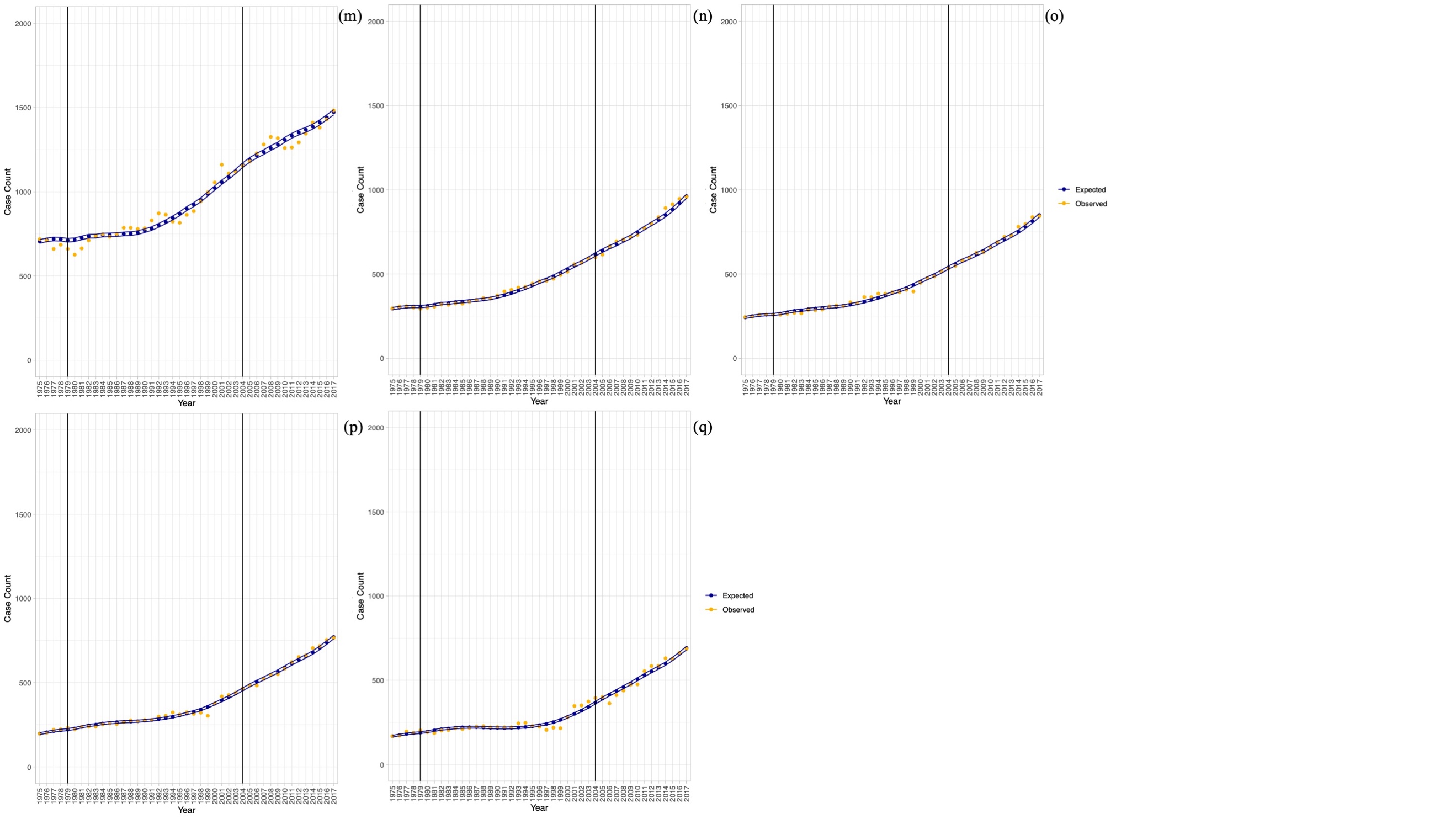
**

**Figure S3. Sensitivity analysis: observed-expected control charts for incidence trends of identified juvenile diabetes algorithms.** (a) algorithm 1: 1+H or 1+P; (b) algorithm 1: 1+H or 2+P; (c) algorithm 1: 1+H or 3+P; (d) algorithm 1: 1+H or 4+P; (e) algorithm 2: 1+H or 1+P; (f) algorithm 2: 1+H or 2+P; (g) algorithm 3: 1+H or 1+P; (h) algorithm 3: 1+H or 2+P; (i) algorithm 1: 1+P; (j) algorithm 1: 2+P; (k) algorithm 2: 1+P; (l) algorithm 2: 2+P; (m) algorithm 2: 3+P; (n) algorithm 2: 4+P; (o) algorithm 2: 5+P

**
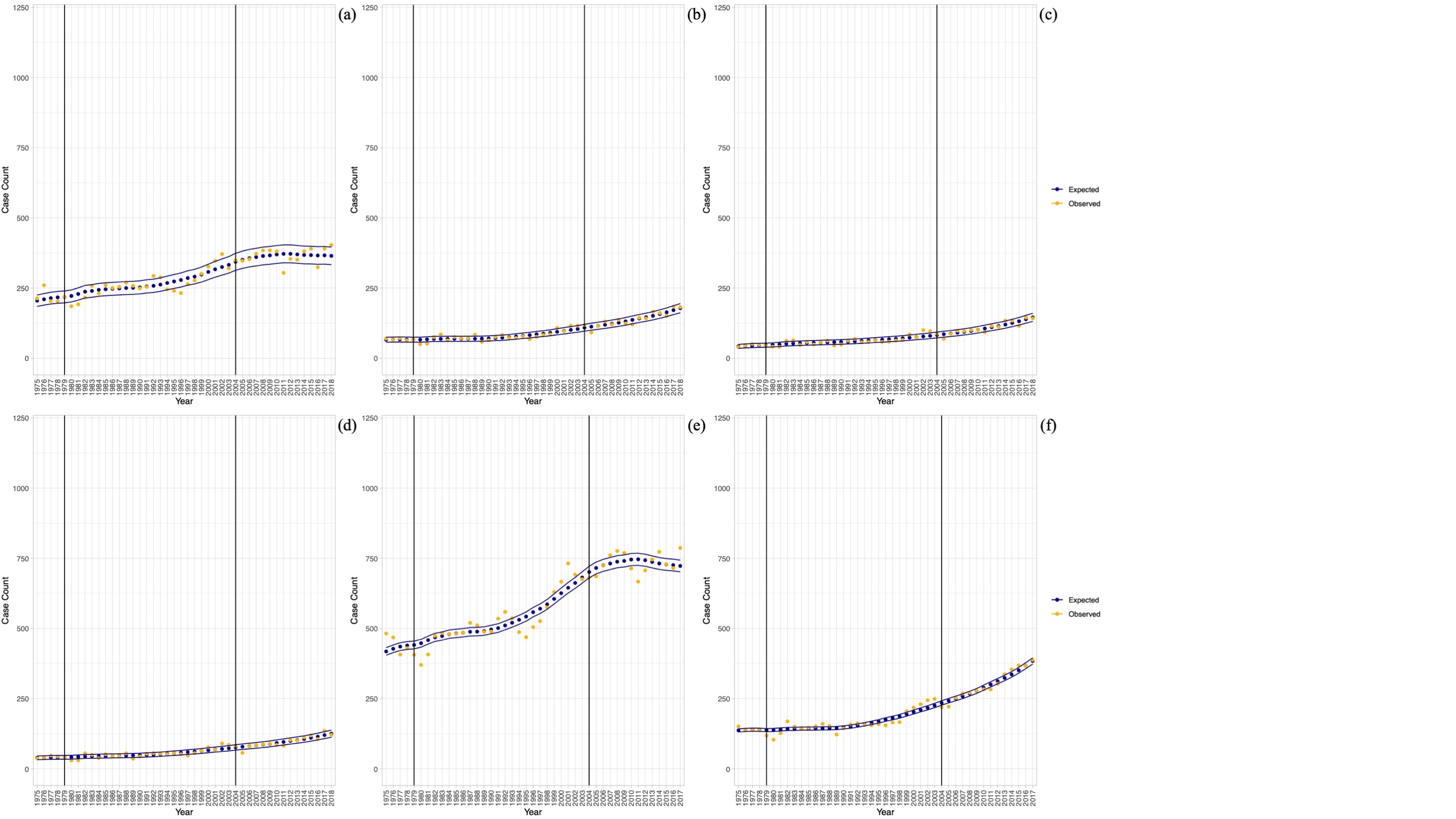
**

**
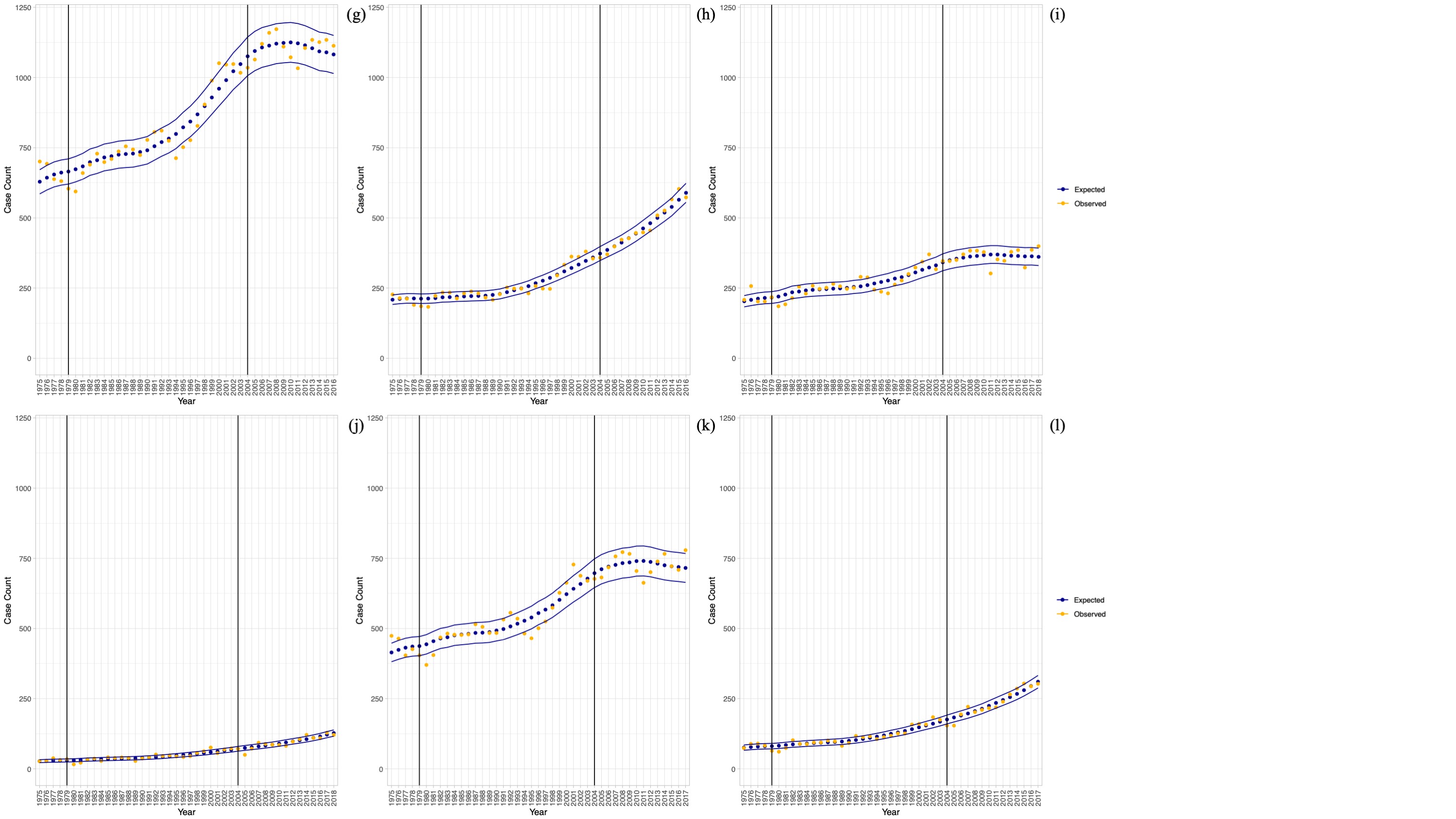
**

**
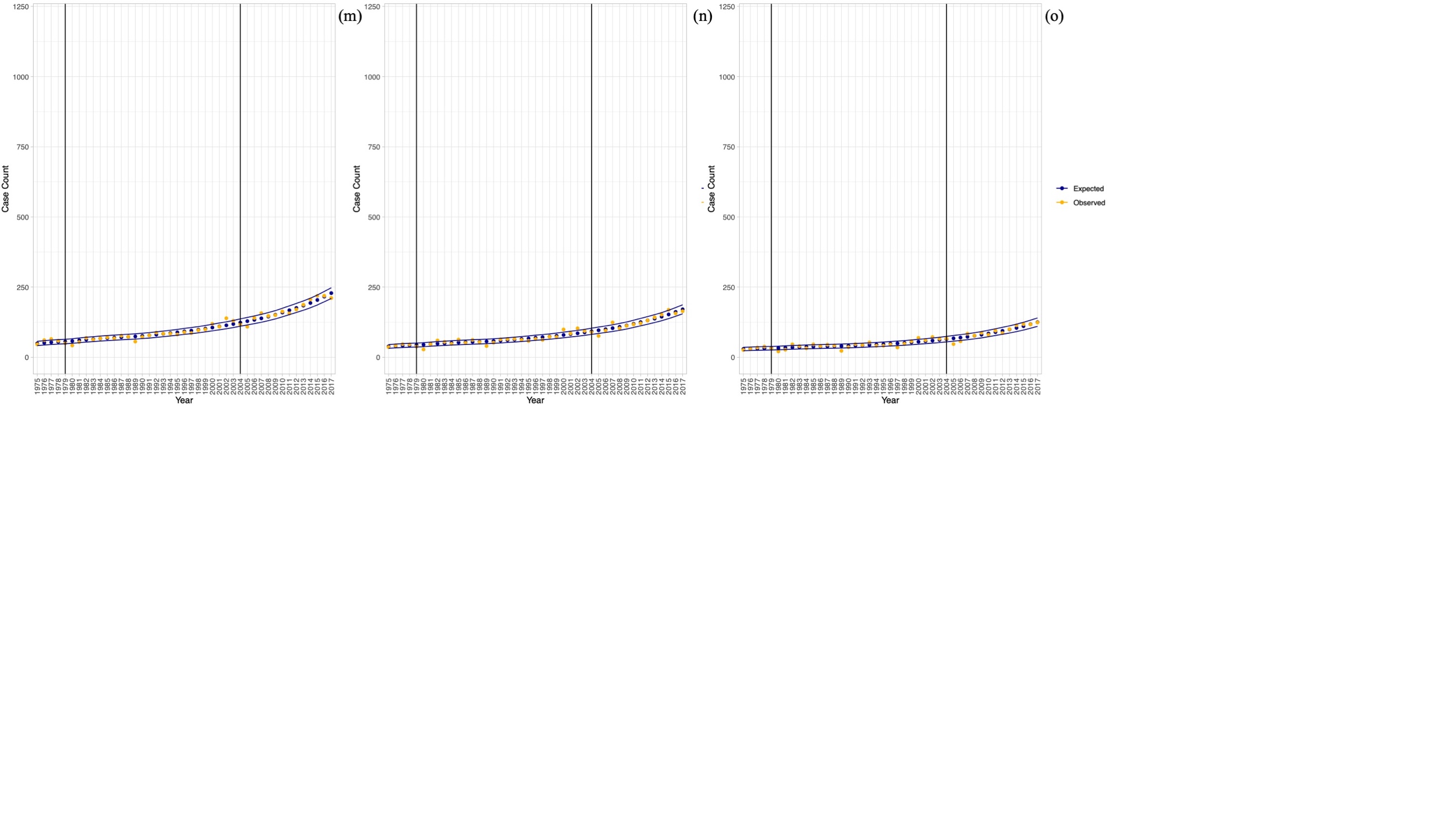
**

**Figure S4. Sensitivity analysis: observed-expected control charts for prevalence trends of identified juvenile diabetes algorithms.** (a) algorithm 1: 1+H or 1+P; (b) algorithm 1: 1+H or 2+P; (c) algorithm 1: 1+H or 3+P; (d) algorithm 1: 1+H or 4+P; (e) algorithm 2: 1+H or 1+P; (f) algorithm 2: 1+H or 2+P; (g) algorithm 3: 1+H or 1+P; (h) algorithm 3: 1+H or 2+P; (i) algorithm 1: 1+P; (j) algorithm 1: 2+P; (k) algorithm 1: 3+P; (l) algorithm 1: 4+P; (m) algorithm 2: 1+P; (n) algorithm 2: 2+P; (o) algorithm 2: 3+P; (p) algorithm 2: 4+P; (q) algorithm 2: 5+P

**
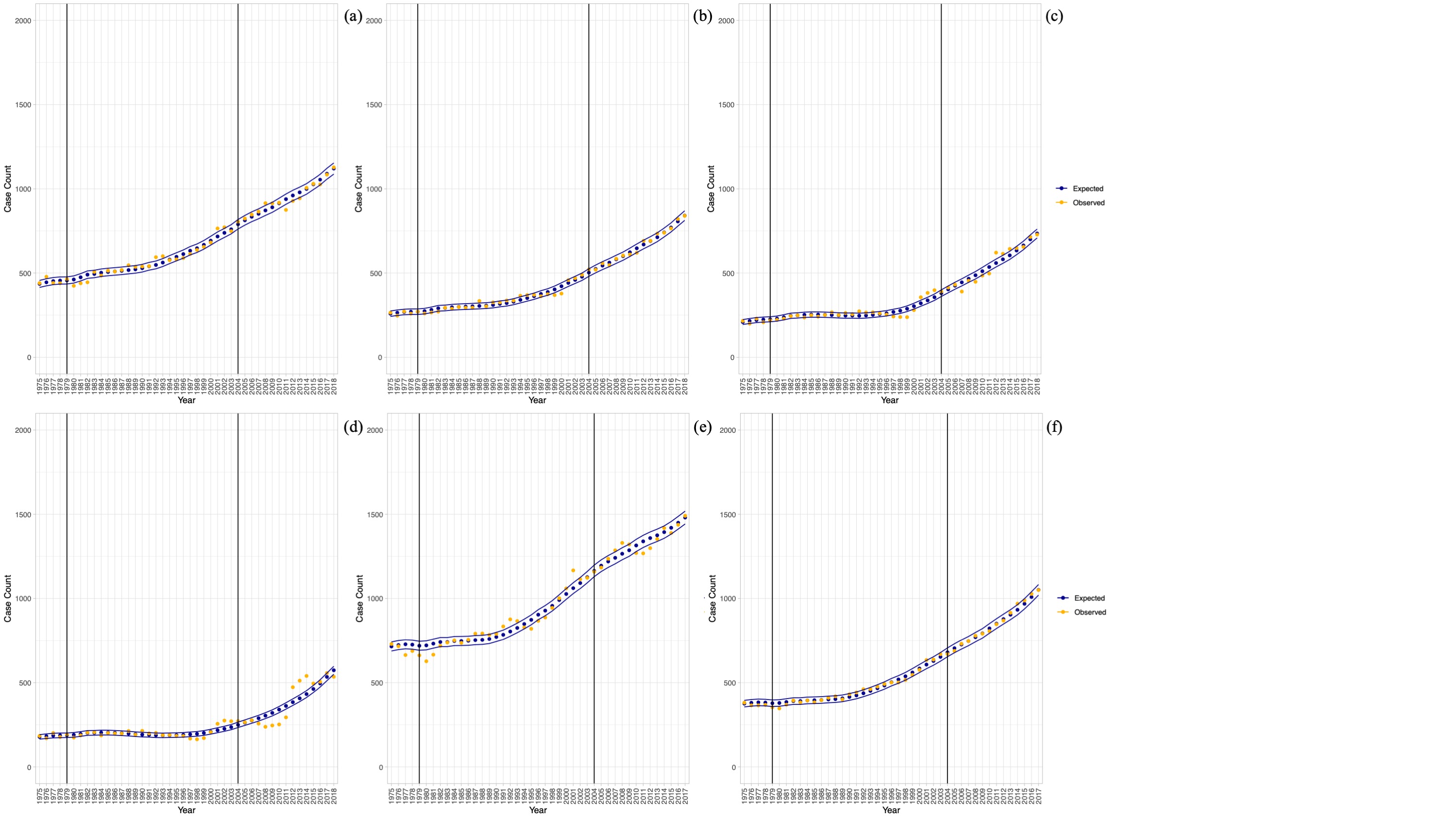
**

**
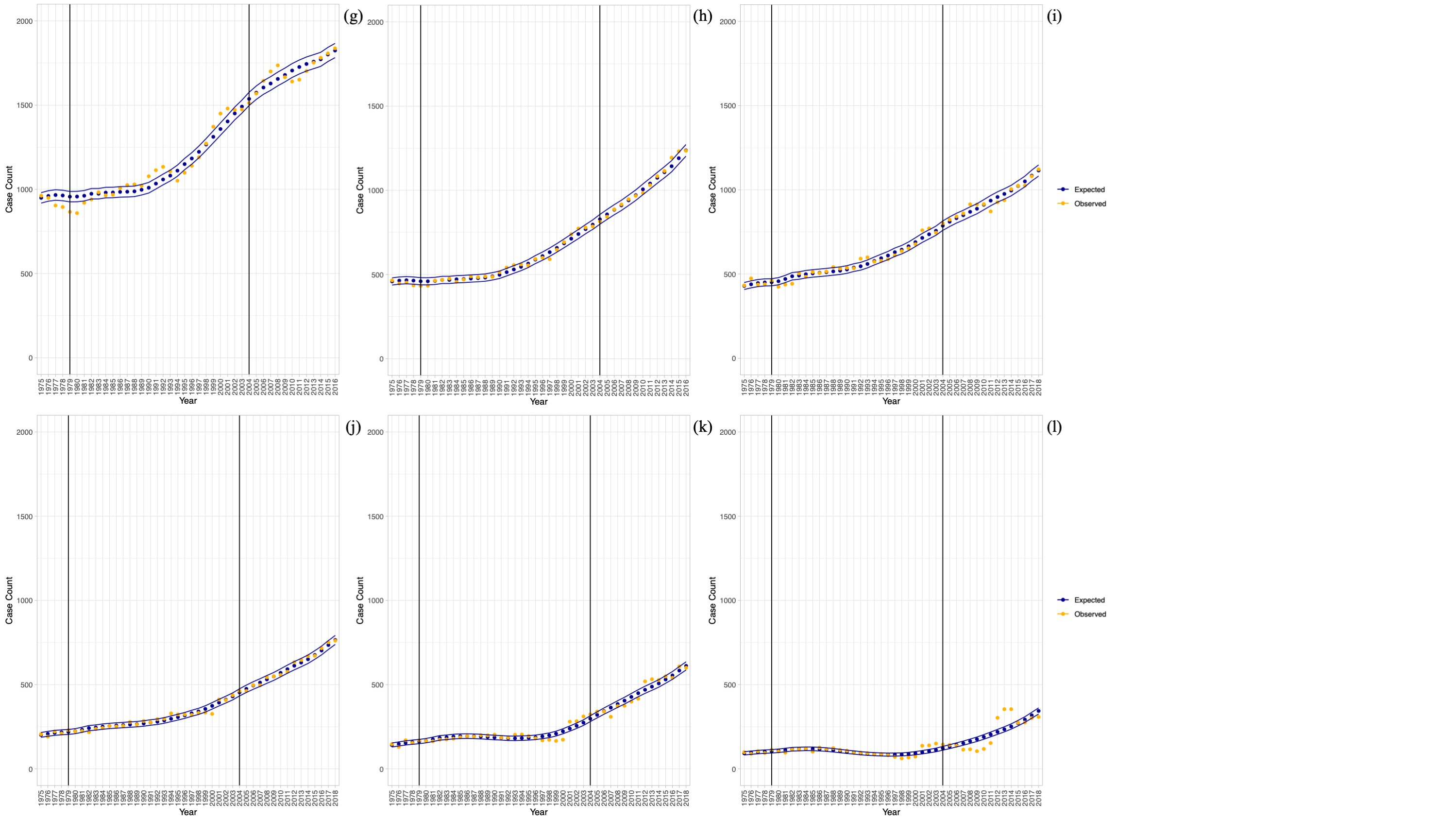
**


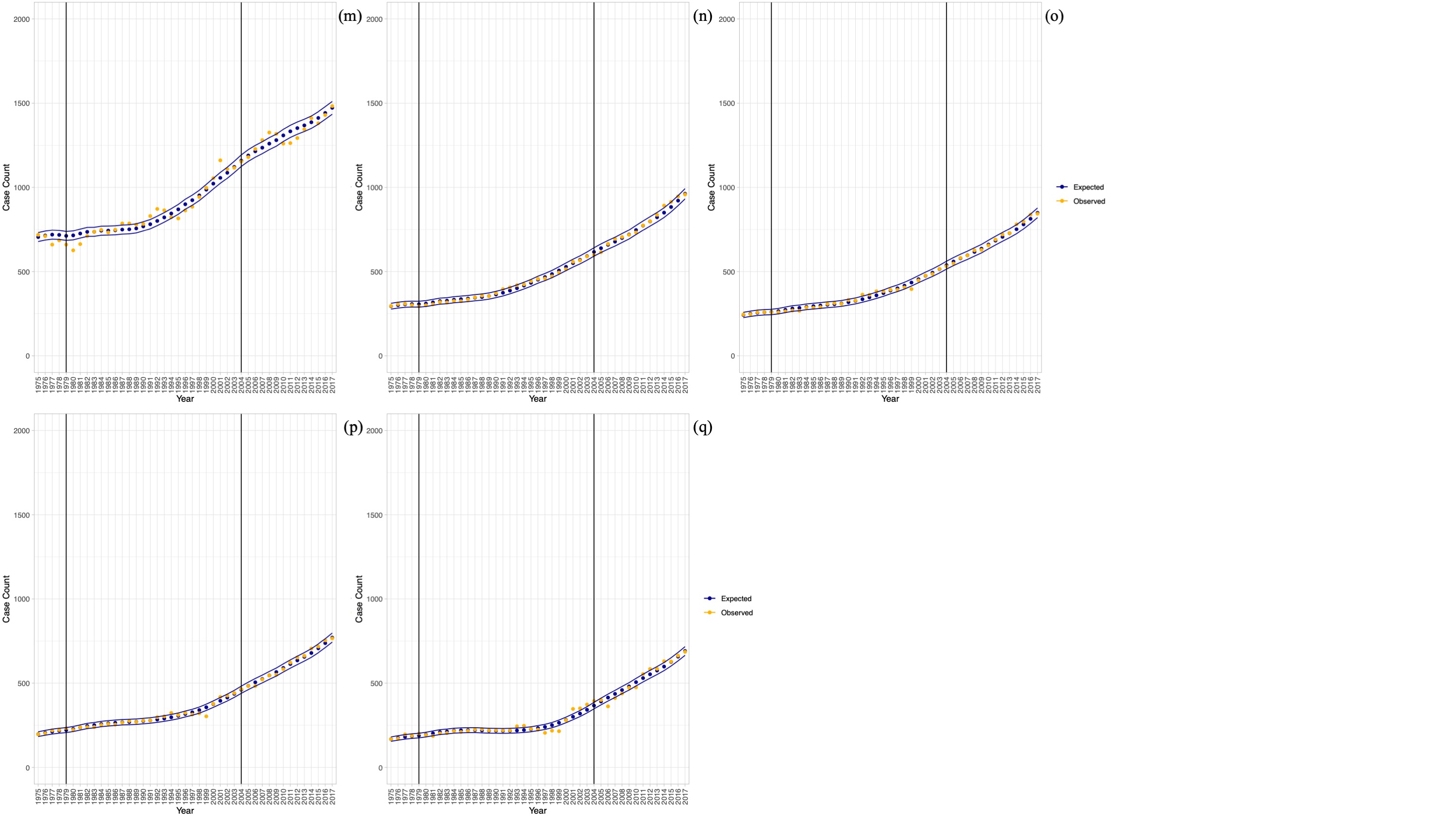


**Figure S5: Sensitivity analysis: algorithm agreement-by-year for out-of-control juvenile diabetes estimates.** Panel (a) shows results for incidence; panel (b) shows results for prevalence.

(a)

**
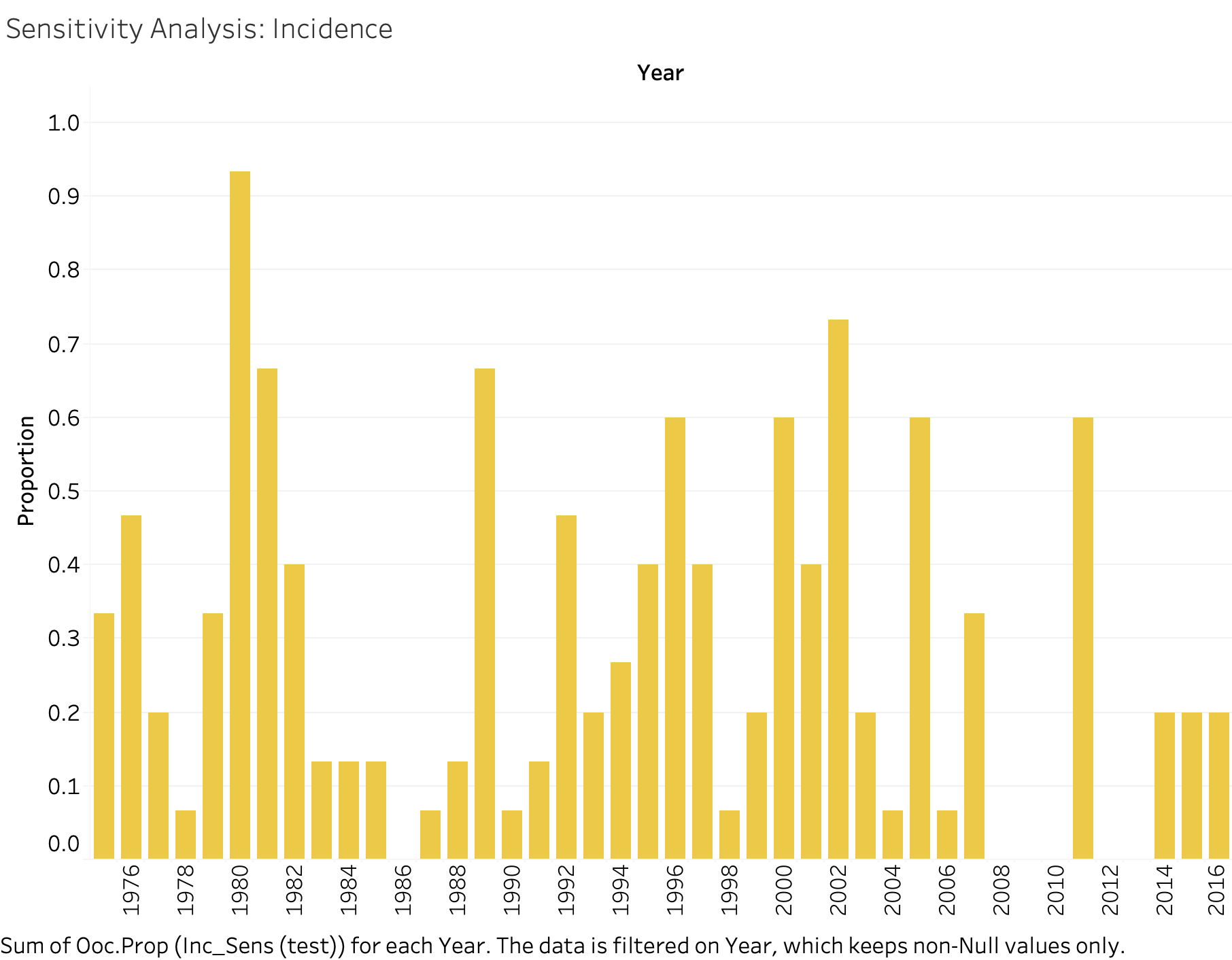
**

Year

(b)


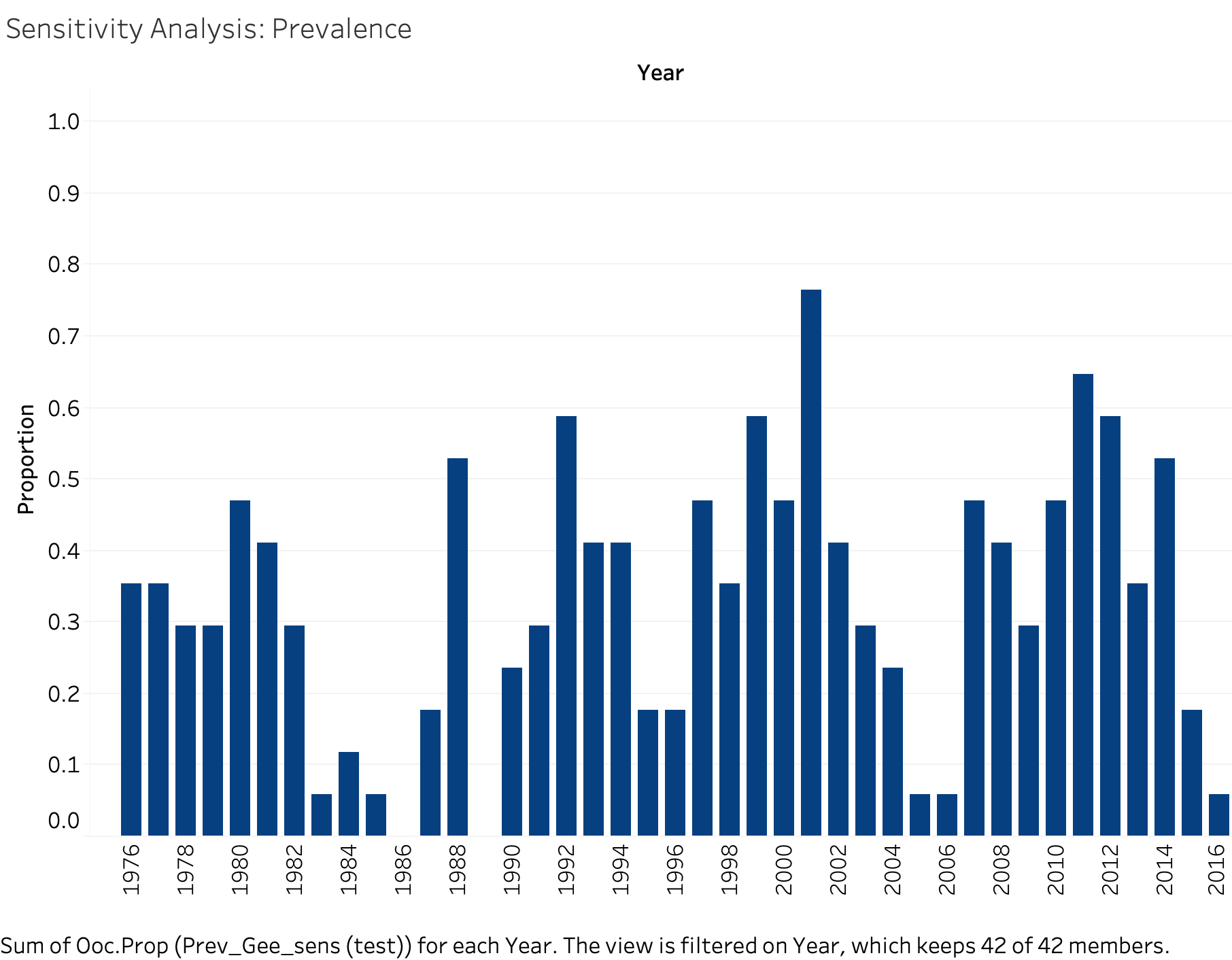


Year
